# Supplementary material for: Examining the responsiveness of the National Health Insurance Fund to people living with hypertension and diabetes in Kenya: a qualitative study
Source: BMJ Open. 2023 Jul 4;13(7):e069330. doi: 10.1136/bmjopen-2022-069330 (PMC10335555; doi:10.1136/bmjopen-2022-069330)
Supplement: Author's manuscript [file bmjopen-2022-069330.draft_revisions.pdf]

BMJ Open is committed to open peer review. As part of this commitment we make the peer review history of every article we publish publicly available.

When an article is published we post the peer reviewers' comments and the authors' responses online. We also post the versions of the paper that were used during peer review. These are the versions that the peer review comments apply to.

The versions of the paper that follow are the versions that were submitted during the peer review process. They are not the versions of record or the final published versions. They should not be cited or distributed as the published version of this manuscript.

BMJ Open is an open access journal and the full, final, typeset and author-corrected version of record of the manuscript is available on our site with no access controls, subscription charges or pay-per-view fees (http://bmjopen.bmj.com).

If you have any questions on BMJ Open's open peer review process please email info.bmjopen@bmj.com

BMJ Open

Examining the Responsiveness of the National Health Insurance Fund (NHIF) to People Living with Hypertension and Diabetes in Kenya: A qualitative study

Journal:	*BMJ Open*
Manuscript ID	bmjopen-2022-069330
Article Type:	Original research
Date Submitted by the Author:	19-Oct-2022
Complete List of Authors:	Oyando, Robinson; KEMRI-Wellcome Trust Research Programme<br>Were, Vincent; KEMRI-Wellcome Trust Research Programme Nairobi, Health Economics Research Unit (HERU)<br>Willis, Ruth; London School of Hygiene and Tropical Medicine Faculty of Public Health and Policy, Department of Health Service Research and Policy<br>Koros, Hillary; Academic Model Providing Access to Healthcare<br>Kamano, Jemima; Moi University, Department of Medicine, School of Medicine, College of Health Sciences,<br>Naanyu, Violet; Moi University, School of Arts and Social Sciences; Moi University College of Health Sciences, Department of Medicine<br>Etyang, Anthony ; KEMRI-Wellcome Trust Research Programme, Department of Epidemiology and Demography<br>Mugo, Richard; AMPATH Kenya<br>Murphy, Adrianna; London School of Hygiene and Tropical Medicine Faculty of Public Health and Policy,<br>Nolte, Ellen; London School of Hygiene & Tropical Medicine, Department of Health Services Research and Policy<br>Perel, Pablo; LSMTM<br>Barasa, Edwine; Centre for Geographic Medicine Research Coast, Health Economics Research Unit
Keywords:	Hypertension < CARDIOLOGY, Health policy < HEALTH SERVICES ADMINISTRATION & MANAGEMENT, Health economics < HEALTH SERVICES ADMINISTRATION & MANAGEMENT, QUALITATIVE RESEARCH, PUBLIC HEALTH

SCHOLARONE™
Manuscripts

I, the Submitting Author has the right to grant and does grant on behalf of all authors of the Work (as defined in the below author licence), an exclusive licence and/or a non-exclusive licence for contributions from authors who are: i) UK Crown employees; ii) where BMJ has agreed a CC-BY licence shall apply, and/or iii) in accordance with the terms applicable for US Federal Government officers or employees acting as part of their official duties; on a worldwide, perpetual, irrevocable, royalty-free basis to BMJ Publishing Group Ltd ("BMJ") its licensees and where the relevant Journal is co-owned by BMJ to the co-owners of the Journal, to publish the Work in this journal and any other BMJ products and to exploit all rights, as set out in our licence.

The Submitting Author accepts and understands that any supply made under these terms is made by BMJ to the Submitting Author unless you are acting as an employee on behalf of your employer or a postgraduate student of an affiliated institution which is paying any applicable article publishing charge ("APC") for Open Access articles. Where the Submitting Author wishes to make the Work available on an Open Access basis (and intends to pay the relevant APC), the terms of reuse of such Open Access shall be governed by a Creative Commons licence – details of these licences and which Creative Commons licence will apply to this Work are set out in our licence referred to above.

Other than as permitted in any relevant BMJ Author's Self Archiving Policies, I confirm this Work has not been accepted for publication elsewhere, is not being considered for publication elsewhere and does not duplicate material already published. I confirm all authors consent to publication of this Work and authorise the granting of this licence.

For peer review only

Examining the Responsiveness of the National Health Insurance Fund (NHIF) to People Living with Hypertension and Diabetes in Kenya: A qualitative study

Robinson Oyando[1], Vincent Were[1], Ruth Willis[2], Hillary Koros[3], Jemima Kamano[4], Violet Naanyu[4,5], Anthony Etyang[6], Richard Mugo[3], Adrianna Murphy[2], Ellen Nolte[2], Pablo Perel[7], Edwine Barasa[1,8]

[1] Health Economics Research Unit, KEMRI Wellcome Trust Research Programme, Nairobi, Kenya
[2] Department of Health Services Research and Policy, London School of Hygiene & Tropical Medicine, London, UK
[3]AMPATH, Eldoret, Kenya
[4] Department of Medicine, School of Medicine, College of Health Sciences, Moi University, Eldoret, Kenya
[5] School of Arts and Social Sciences, Moi University, Eldoret, Kenya
[6] Department of Epidemiology and Demography, KEMRI-Wellcome Trust Research Programme, Kilifi, Kenya
[7] Department of Non-Communicable Disease Epidemiology, London School of Hygiene and Tropical Medicine, London
[8] Center for Tropical Medicine and Global Health, Oxford University, Oxford, 01540, UK

Corresponding author:

Robinson Oyando
Health Economics Research Unit, KEMRI Wellcome Trust Research Programme,
P.O.BOX 43640-00100,
Nairobi, Kenya
Email: ROyando@kemri-wellcome.org

For peer review only

For peer review only - http://bmjopen.bmj.com/site/about/guidelines.xhtml

ABSTRACT

Objectives: To assess the responsiveness of the National Health Insurance Fund (NHIF) Supa Cover benefit package to the needs of individuals with diabetes and hypertension in Kenya.

Design, setting and participants: We carried out a qualitative study and collected data using key informant interviews (n = 39) and focus group discussions (n = 4) in two purposively selected counties in Western Kenya. Study participants were drawn from NHIF officials, county government officials, health facility managers, healthcare workers and individuals with NCDs who were enrolled in NHIF. We analysed data using thematic analysis.

Results: Study participants reported that the NHIF Supa Cover benefit package expanded access to services for people living with NCDs. However, the NHIF members and healthcare workers had inadequate awareness of the NHIF service entitlements. The NHIF benefit package inadequately covered the range of services needed by people living with NCDs and the benefits package did not prioritise preventive and promotive services. Sometimes NCD patients were discriminated against by healthcare providers who preferred cash-paying patients, and some NHIF-empanelled health facilities had inadequate structural inputs essential for quality of care. Study participants felt that the NHIF premium for the general scheme was unaffordable, and NHIF members faced additional out-of-pocket costs because of additional payments for services not available or covered.

Conclusion: Whereas NHIF has reduced financial barriers for NCD patients, to enhance its responsiveness to patient needs, NHIF should implement mechanisms to increase benefit package awareness among members and providers. In addition, preventive and promotive services should be included in NHIF's benefits package and mechanisms to monitor and hold contracted providers accountable should be strengthened.

Keywords: Universal health coverage, non-communicable diseases, reforms, Kenya, Low- and Middle-Income Countries

For peer review only - http://bmjopen.bmj.com/site/about/guidelines.xhtml

Strengths and limitations of this study

- This study used in-depth interviews and focus group discussions to comprehensively examine how responsive the national health insurance fund is to the healthcare needs of patients with chronic conditions.
- The study recruited participants from both urban and rural sites and health staff from a range of cadres and thus provides evidence from a wide range of respondents with varied experiences.
- Including chronic disease patients who had discontinued membership from the national health insurance scheme could have yielded richer findings on the scheme's responsiveness.

For peer review only

INTRODUCTION

Non-communicable diseases (NCDs) account for the greatest share of the global disease burden and are one of the significant health challenges of the century [1, 2]. Low-and-middle income countries (LMICs) bear the highest NCD burden [3]. While infectious diseases contribute to the highest share of disease burden in sub-Saharan Africa (SSA), the burden of NCDs is rapidly increasing and is expected to grow [4]. The latest estimates from the Global Burden of Disease study suggest that the Disability Adjusted Life Years for NCDs have increased by 13.1% between 1990 and 2019 [5]. Responsive health systems provide high-quality health services equitably and efficiently to the population in need without the risk of catastrophic or impoverishing healthcare expenditures [6, 7]. Evidence suggests that most NCD patients in LMICs do not have equal access to NCD services such as screening and treatment [8-13]. Among other things, this is due to lack of access due to financial barriers and inadequate capacity of health facilities to offer NCD care [8, 12-14].

Global efforts to address the growing burden of NCDs are reflected in the 2030 Sustainable Development Goals (SDG) [15]. One of the mechanisms to enable financial access to NCD services

For peer review only - http://bmjopen.bmj.com/site/about/guidelines.xhtml

is designing and implementing pre-payment health financing mechanisms from tax or compulsory insurance contributions that provide financial risk protection to households and individuals living with NCDs and enhance access to quality NCD services [16, 17]. Some evidence suggests that having health insurance is beneficial in enabling access to NCDs services [18-20]. Nevertheless, health insurance coverage in SSA remains low, with only four out of 36 SSA countries having health insurance coverage of any type exceeding 20% [21]. In addition, tax-based pre-payment mechanisms are preferred as an equitable health financing mechanism in SSA given the context of a large informal sector with a low contributory capacity [22].

In Kenya, NCDs account for 50% of inpatient admissions and 55% of hospital deaths [23]. Evidence suggests that NCDs pose a major economic burden to households in Kenya [24-27]. Besides, there is a mismatch between the need and supply of cost-effective interventions such as screening and treatment for NCDs like hypertension and diabetes, with the poor being disproportionately disadvantaged [13]. To address these and broader health systems challenges, the government of Kenya has implemented several policy reforms to move the country towards UHC by 2030 [28-31]. One such reform is the policy decision to use the NHIF as one of the key pre-payment health financing mechanisms [32]. Established in 1966, NHIF is a public insurer in Kenya that provides mandatory health insurance to all Kenyans [28]. However, national NHIF coverage remains low, at 16% [33]. In the past 11 years, several reforms have been introduced in NHIF in an effort to enhance its capacity to deliver UHC [28]. Among these reforms is the broadening of the benefit package ('Supa Cover') of its general scheme (that targets all Kenyans) to include a range of services for NCDs, among others [28]. In addition to coverage for general inpatient and outpatient services, the expanded benefits package includes a range of what is labelled specialised services that include those for NCDs [34].

Kenyan citizens including those that work in both the formal and informal sectors are eligible to enrol to the NHIF's national scheme [35]. Membership in this scheme requires a monthly premium that is graduated according to employees' salary level for the formal sector and is a fixed rate (KES 500 per month) per household for individuals in the informal sector or those receiving subsidies from the government (the poor and vulnerable) [28, 29]. For the latter, the government funds their premiums under several programs dubbed "sponsored programs". These include the health insurance subsidy program for the poor (HISP), and the insurance subsidy program for the disabled and elderly [28, 29]. The NHIF contracts public, private for-profit and private not-for-profit healthcare facilities to provide services to NHIF members and pays them using a range of provider payment mechanisms [32, 34]. NCD care services under the Supa Cover benefit package are

For peer review only - http://bmjopen.bmj.com/site/about/guidelines.xhtml

reimbursed using case-based mechanisms, as well as capitation for outpatient services, and per admission day rate for inpatient services [34].

This study is part of a larger study that seeks to inform and support the scale-up of the Primary Health Integrated Care Project for Chronic Conditions (PIC4C) model for integrated management of people with hypertension, diabetes, and breast and cervical cancers in Kenya [36]. Alongside other work packages in the larger study, our work package has three overarching aims: i) to measure the effectiveness of the NHIF national scheme benefit package to provide financial risk protection to individuals with chronic diseases; ii) to examine the extent to which the NHIF national scheme benefit package is responsive to the needs of individuals with and hypertension; and iii) to examine how the provider incentives generated by provider payment arrangements of the NHIF national scheme benefit package influence equity, efficiency, and quality of care.

There is limited evidence about the degree to which the expanded scheme is addressing the needs of people with NCDs in Kenya. This study aims to generate evidence that would be relevant in informing policymakers and other stakeholders on how the configuration of health insurance reforms can be tailored to address the needs of NCD patients and thus contribute to the achievement of international benchmarks for NCDs spelt out in the Sustainable Development Goals.

METHODS

Study setting

Kenya is a lower-middle income (LMIC) country located in the East Africa region [37]. There are two levels of administration in Kenya's health system: national and county [38]. The national government (Ministry of Health) is responsible for policy and regulation in the health system while the 47 county governments are responsible for service delivery [39]. The Kenya health system is pluralistic, with an almost equal share of service provision by the public and private providers [40].

Kenya's health system has a four-tiered structure. Tier 1 is at the community level where community-based healthcare demand creation activities are undertaken as stipulated in the community health strategy; Tier 2 is at the primary care level where preventive and promotive services are provided in dispensaries and health centres; Tier 3 consists of both primary and secondary referral hospitals; and Tier 4 consists of tertiary referral hospitals under the direct management of the Ministry of Health (MoH) [41].

The sources of health financing in Kenya include out-of-pocket (OOP) payments by households, government (national and county) tax revenues, donors, and member contributions from NHIF

For peer review only - http://bmjopen.bmj.com/site/about/guidelines.xhtml

and private insurance companies [42, 43]. For instance, in the financial year (FY) 2015/16, current health expenditure (CHE) as a percentage of gross domestic product was 5.2%, CHE per capita in US$ was 78.6 while the proportion of CHE financed through OOP payments was 26.1% [42]. In addition, the total health expenditure (THE) on NCDs as a proportion of THE was 11% in FY 2017/18 [44].

For peer review only

For peer review only - http://bmjopen.bmj.com/site/about/guidelines.xhtml

Study sites

This study was carried out in Busia and Trans Nzoia counties in Western Kenya, where Moi University, Moi Teaching and Referral Hospital through AMPATH (Academic Model Providing Access to Healthcare) have partnered for several years with county governments to strengthen health systems across various care levels [45]. Table 1 outlines the key characteristics of the two counties. Launched in 2018, the PIC4C model is implemented by a partnership between AMPATH/Moi, the MoH and the World Bank and includes (i) early case finding of people with hypertension, diabetes, cervical/ breast cancer at service level 1; (ii) structured referral to service providers at level 2 for confirmation of diagnosis and treatment initiation or referral to level 3 or 4 using structured protocols; (iii) initiation of treatment using structured treatment protocols and decision support tools at levels 2, 3 and 4; (iv) retention of patients in care supported by ongoing training of health workers at all care levels; (v) monitoring and evaluation supported by a health information system; and (vi) advising patients in care to enrol in the NHIF national scheme for sustainable health financing [36].

For peer review only

Table 1 Characteristics of the study setting

**Characteristics**	**Busia county**	**Trans Nzoia county**
Population‡	893,681	990,341
% Adults (18+ years) ‡	52%	53%
% Females ‡	52%	51%
Life expectancy at birth	47 years	60.5 years
Poverty level	64%	50%
Estimated hypertension prevalence	22%	27%
Estimated diabetes prevalence	1.5%	2%
NHIF coverage	12%	11%
Number of health facilities†	184 community units served by 47 dispensaries, 12 health centers, 5 sub-county and 1 county hospital	198 community units served by 38 dispensaries, 8 health centers, 6 sub-county health facilities and 1 county hospital
Number of patients with selected NCDs currently being serviced in AMPATH sites	6,060 patients with hypertension, 1,113 with diabetes and 200 with cervical cancer.	4,375 patients with hypertension, and 2,089 with diabetes.
Number of facilities where the PIC4C pilot was implemented‡‡	40 facilities (5 subcounty hospitals, 12 health centers and 23 dispensaries).	33 facilities (6 subcounty hospitals, 8 health centers and 19 dispensaries).

(Sources: ‡‡[36]; ‡[46]; †[47])

For peer review only - http://bmjopen.bmj.com/site/about/guidelines.xhtml

Study conceptual framework

This study's conceptual framework (Figure 1) assumes that for the NHIF to be effective in providing financial risk protection to NCD patients and their households while enhancing access to quality health services, it is imperative that 1) the benefit package represents services that patients need 2) that patients actually access the entitlements from the benefit package, and 3) the NHIF reimbursements to healthcare providers adequately covers the fees providers charge patients for services (depth of cover). Therefore, following the study's conceptual framework, responsiveness in this study is conceptualised to mean that the NHIF Supa Cover meets the needs of NCD patients in each of the three elements.

Insert Figure 1 here

Study design and data collection

We conducted a qualitative study where we interviewed purposively selected participants. We conducted four focus group discussions (FGDs) with NCD patients in Busia (1 rural and 1 urban) and Trans Nzoia (1 rural and 1 urban) counties who were enrolled in the NHIF national scheme. FGD participants were selected from an existing PIC4-C scale-up study database (matched cohort wave 2).[36] FGD participants were eligible if 1) they were actively enrolled in NHIF at the time of recruitment, 2) they had either diabetes or hypertension or comorbid (both diabetes and hypertension), 3) were more than 18 years of age, and 4) if they consented to participate in the study. Eligible participants were invited to the FGD sessions by telephone. FGD sessions took place at the nearest public health facility and at a time convenient to the participants. In-depth interviews (IDIs) were held with county health officials (n = 7), county NHIF branch officers (n = 3), health facility managers (n = 12), and front-line healthcare workers (n = 17) composed of medical officers, pharmacists, nursing officers and clinical officers. All study participants were consented and signed informed consent forms before participating in the interview sessions. FGD and IDI sessions were facilitated by two trained qualitative researchers. The FGDs were facilitated in Kiswahili while the IDIs were conducted in English. With consent from the participants, the sessions were audio recorded and complimentary notes were taken. FGDs lasted for an average of 48 minutes while the interviews lasted for an average of 35 minutes. Table 2 outlines the number of respondents interviewed by their respective categories.

For peer review only - http://bmjopen.bmj.com/site/about/guidelines.xhtml

For peer review only

Table 2 Summary of Respondents

County	Respondent	No. interviewed
Busia	County officials	4
	NHIF branch officers	1
	Facility managers	6
	Frontline healthcare workers	10
	NCD patient FGDs	2 (1 urban [7 participants]; 1 rural [6 participants])
Trans Nzoia	County officials	3
	NHIF branch officers	2
	Facility managers	6
	Frontline healthcare workers	7
	NCD patient FGDs	2 (1 urban [7 participants]; 1 rural [5 participants])

The FGDs and IDIs were conducted using semi-structured topic guides. The development of the questions in the discussion and interview guides was informed by the study's conceptual framework (Figure 1). The questions focused on NCD services that NHIF pays for, adequacy of the range of NCD services covered by NHIF, participants' experience with NCD service provision, quality of NCD care, prioritisation of preventive and promotive services in the NHIF Supa cover scheme, NHIF provider payment mechanisms, strengths, and weaknesses of the NHIF national scheme. At the end of each session, the facilitator and note taker held debrief sessions which facilitated improving the interviewing process and revision of some questions to enhance clarity and inclusion of emerging themes that needed further exploration. Data collection was discontinued when data saturation was attained. Data were collected between June and July 2021.

Data management and analysis

Qualitative FGDs and IDIs audio recordings were transcribed verbatim and translated to English where necessary. To validate and familiarise ourselves with the transcribed transcripts, we read and re-read the transcripts and ensured correctness by re-listening to sections of the audio recordings. Validated transcripts were imported into NVivo QSR 12 for coding and analysis. We used a thematic approach to analyse the qualitative data. Thematic analysis involved familiarisation with the data through reading and re-reading the transcripts to identify emerging ideas, meanings, patterns and potential themes [48]. In the first stage of analysis, two authors (RO and RW) working independently coded the first few transcripts to identify key themes and developed the initial

For peer review only

coding framework. This involved the generation of initial codes, grouping developed codes into descriptive themes by identifying matching patterns. Thereafter, a discussion among three authors (RO, RW and EB) was held where individual codes were compared, and a final coding framework was arrived at by consensus. Whereas the coding of the transcript was guided by the study's conceptual framework, as data coding progressed iteratively, the coding framework was updated based on the emerging codes. In the next step, RO charted the data. During charting, emerging themes are identified through a process where coded data is reorganised. In this stage, RO summarised the ideas from each code with illustrative quotes from the data. EB checked and refined coding charts developed by RO. In the final phase, summaries from each thematic heading were critically interpreted and synthesised to identify key policy-relevant findings.

Ethical considerations

This study was reviewed and approved by the Institutional Ethics and Research Committee of Moi University (IREC 0003586) and the London School of Hygiene and Tropical Medicine (17940).

Patient and public involvement statement

The roll out of the PIC4C model in the two counties was accomplished by involving village chiefs and elders in the process. Patients and members of the public were not involved in the development of the proposal, or the design of the research presented here.

For peer review only

For peer review only - http://bmjopen.bmj.com/site/about/guidelines.xhtml

RESULTS

We conducted a total of 39 IDIs (17 men and 22 women) and four FGD sessions with 28 participants (15 women and 13 men). Six FGD participants had diabetes, 13 had hypertension and nine had both diabetes and hypertension. The results are presented under five key thematic areas: access to NCD services, awareness of NCD service entitlements in the NHIF Supa Cover scheme, adequacy of the range of NCD services covered by NHIF, provider payment mechanisms for NCD services and affordability of NHIF Supa Cover scheme. Summaries of key findings are provided under each thematic area.

Access to NCD Services

The NHIF benefit package expanded access to services for people living with NCDs by reducing financial barriers. Several participants reported that the NHIF package had facilitated access to a range of NCD care services that they would otherwise have had to pay for OOP. Services that were reported to be covered included inpatient care and referral to NHIF accredited healthcare facilities, and medications. For example, one patient noted:

"When I have the NHIF, I have hope that I can go to the hospital even if I don't have any money and I can get medication compared to the one who does not have the NHIF." Rural FGD Participant, County A

One healthcare worker specifically related NHIF coverage to increased clinic attendance:

"The strength of NHIF super cover is that it has alleviated the suffering these patients. There are patients who had stopped coming to the clinic because of financial challenges, but with the NHIF cover, we found that almost all our patients are always coming for the clinic; those who are covered are always coming for the clinic without fail." Healthcare worker, Sub-County Hospital, County A

Being enrolled in NHIF was reported to have beneficial implications for patients' wider financial situations, for example, avoiding the need to borrow money or sell assets to pay for health care services.

"It prevents you from borrowing and reduces stress for you and even respect comes. It prevents you from selling the farm, animals and any other thing." Rural FGD Participant, County B

Further, financial barriers were reduced for all population groups because the NHIF did not limit the enrolment of individuals on age or pre-existing NCD condition, unlike private health insurance.

For peer review only

Awareness of NCD service entitlements

Some NHIF members and healthcare workers had inadequate awareness of the NHIF service entitlements. While some study respondents showed awareness of the NCD care services that are covered by the NHIF Supa Cover scheme, others indicated that they were not adequately aware of service entitlements under the scheme.

"If you were to get a patient who needed radiotherapy or chemotherapy in my facility, we don't offer those services. I don't know if NHIF caters for that. I don't know to what extent… it does not cover some services such as chemotherapy or radiotherapy. I don't know if it caters for them, am not sure. I hope it does." Healthcare worker, Sub-County Hospital, County A

Inadequate awareness of NCD service entitlements in the Supa Cover scheme resulted in some NCD patients being wrongfully turned away by healthcare workers.

"I asked if the NHIF card can be used to do x-ray and whoever was there told me that it cannot, you have to go outside the hospital. I didn't go and that is when I realised that the NHIF can do x-ray. That is when I realised that there are others who get services while others do not. The last time there was a nurse who came and asked on my behalf and she was told that it was working, and I got the services." Urban FGD Participant, County B

Adequacy of the range of NCD services

The NHIF benefit package inadequately covered the range of services needed by people living with hypertension and diabetes. While the cover catered for outpatient consultation and inpatient admissions, there was a strong perception among most NHIF members and healthcare workers that the NHIF Supa Cover package did not adequately cover the range of diagnostic, monitoring, and medicines that they needed and did not explicitly cover NCD medicines. Several participants reported incidents of relevant services having to be covered OOP by service users themselves.

"One day I came here. They sent me to go get an x-ray. When I got there, I was told that they cannot cover for that expense. Go out. When I got out there, I asked and was told KES 5,200/= to get the x-ray. I was perplexed." Urban FGD, County A

"First thing urinalysis, HbA1c [glycated haemoglobin] *and UEC* [urea, electrolytes and creatinine]. *UEC will guide us whether to start with insulin oral or metformin alone. Then afterwards you need to follow up on this patient after three months you need HbA1c, but you find most of our clients mostly those relying on insurances, they're not able to pay so I can comfortably say they are not adequately covered using NHIF."* Medical Officer, Private Hospital, County A

For peer review only - http://bmjopen.bmj.com/site/about/guidelines.xhtml

The NHIF benefit package did not prioritise preventive and promotive services for NCDs. Some study respondents felt that the NHIF Supa Cover does not prioritise preventive and promotive services such as screening for NCDs but instead prioritises curative care after a patient is diagnosed with hypertension or diabetes. It was further felt that the NHIF prioritised secondary and admission care in hospitals rather than primary care services at lower-level facilities.

"The Supa Cover mostly deals with curative aspects of treatment. So, it doesn't deal with preventive aspects of treatment. So, it waits for a client to fall sick, then they can get services. There is no such thing as a preventive service, maybe going for annual check-up unless of course, you are covered under the civil servant type of cover where you have access to one check-up per year." Facility Manager, Sub-County Hospital, County A

"We were told that NHIF is able to cater for health checks, routine screening. And unfortunately, that one is not being done for everybody; it is not for the Super cover, it is for these other packages." County Official

Provider payment mechanisms for NCD services

The design of the outpatient capitation payment mechanism reduced geographical access to services. The capitation payment mechanism required patients to be registered to a specific health facility for outpatient services. Some participants mentioned that this limited access to outpatient services when patients travelled far from their registered facility, and hence compromised continuity of care.

"The government should look into that… [so that] *if you have NHIF, you can receive treatment anywhere. It is not a must that one chooses a hospital to receive treatment."* Rural FGD Participant, County A

Quality of NCD care

NHIF patients were sometimes discriminated against by healthcare providers. Some study participants felt that cash-paying patients received more attention and care from healthcare workers than NCD patients enrolled in NHIF. For instance, respondents living with NCDs observed that healthcare workers did not give them the support they required when in a health facility once they mentioned that they had NHIF cards.

"When you walk in the corridors and meet a doctor and you tell them you have NHIF card, they look at you differently. It is like people see you as desperate because you are mentioning that card. I think

For peer review only - http://bmjopen.bmj.com/site/about/guidelines.xhtml

they prefer cash because if you say you have the card, they don't want to assist you." Urban FGD Participant, County B

"There are times when you waste time walking around the hospital with no help until you decide to use cash instead of wasting the whole day. Sometimes they have a negative attitude if you talk to them about the card." Urban FGD Participant, County B

However, other participants had dissenting views as they reported that there was no difference in how healthcare workers handled NCD patients enrolled in NHIF and those who are not.

"No favouritism is there because one is using NHIF and the other one is using cash." Rural FGD Participant, County B

Some patients related how they were sometimes denied the healthcare services they needed because they had an NHIF card. These patients were instead asked to seek services out of the hospital.

"I was just given Nifedipine alone for blood pressure. I had my fellow patient who was given all the drugs because she paid cash. She was given all drugs. I left there asking myself what the card had done. I was discharged and told to go and buy drugs. I was given only one drug." Urban FGD Participant, County B

Some NHIF-empanelled health facilities had an inadequate structural quality of care. This was characterised by overcrowding at the clinics, erratic supply of NCD medicines, diagnostic supplies and equipment. Some participants reported that they had to return on another clinic day or seek services in other (often private) health facilities and incur OOP.

"Again, after you've come for clinic, there is a schedule, and patients are many. You are told that time is up for receiving your patient book. Yet you got delayed at the laboratory. I have seen others being returned. Their book gets rejected." Urban FGD Participant, County A

"The challenge that is there, it is like there are some tests that they don't do. Like today I did ECG [electrocardiogram], to know the condition of my heart because the other day I came to the hospital. I told the doctor that I had pain that I could not understand. Then I was told to come and do ECG. The machine for ECG was spoilt and because I wanted the service, I had to go outside the hospital, and I paid." Urban FGD Participant, County B

Some patients and healthcare workers reported that public health facilities often did not provide inpatient diets that were suitable for patients with hypertension and diabetes. The meals they

For peer review only - http://bmjopen.bmj.com/site/about/guidelines.xhtml

were served during inpatient admission in public facilities were contrary to the dietary advice they were given to manage their conditions.

"If they prepare *uji* (porridge), it has a lot of sugar. And you have been told not to take sugar. Who will prepare yours now? If they knew that, they would set apart a ward." Rural FGD Participant, County A

Affordability and Financial Risk Protection

Study respondents felt that the NHIF premiums for the general scheme were unaffordable. The KES 500 per month premium was deemed to be beyond reach by some respondents, especially in rural areas. As a result, people living with NCD could not keep up with the premium payments and regularly defaulted.

"The NHIF should reduce the premiums since the people in the rural areas see KES 500 as a lot of cash and fail to register or remain active." Rural FGD Participant, County B

Furthermore, individuals from the informal sector (whose membership was voluntary) who needed to undergo costly or complicated procedures were required to pay their monthly premium subscription at least one year in advance before NHIF authorised them to undergo any procedure. This was deemed unaffordable by many respondents.

"NHIF members from the informal sector are normally required to pay premiums at least more than 2 years in advance, depending on the cost of the procedure that the person is required to do. They are required to pay premiums for 2 years or more before the preauthorisation can be approved." Healthcare worker, FBO, County A

"If you're a NHIF contributor and self-employed, you pay the KES 500 package every month. You cannot get the surgery done unless you get the pre-authorised form, and you should have paid premiums upfront for 1 year for the surgery to be approved. You will find that most of these patients are old, some of them are unemployed so while they can pay KES 500 per month, it becomes a very big issue to pay for a year or more." County Official, County A

NHIF members faced additional out-of-pocket costs because of administration charges, input supply shortages, balance billing, and payments for services not covered. Due to supply-side inefficiencies, some patients reported that they were required to make OOP to purchase consumables like gloves or photocopying fees for receipts.

"Everyone paying for NHIF is struggling. Like the other day on Wednesday. The day has come, we get there, KES 10/= for photocopy and KES 50/= for gloves." Rural FGD Participant, County B

For peer review only - http://bmjopen.bmj.com/site/about/guidelines.xhtml

Further, the reimbursements NHIF paid providers for inpatient and outpatient services were deemed insufficient to cover the cost of offering the services. Hence, patients had to make additional payments to bridge the gap in service delivery costs.

"Per day NHIF payment for inpatient is KES 1,500, while outpatient capitation is KES 1,200 so they can actually surpass. In a day the bill can actually go beyond that because we are looking at for example cost of insulin, the cost of the drugs they are getting… So, in as much as NHIF would pay the KES 1,200, now the patient has to top up, yes." Facility Manager, County Referral Hospital, County A

Services that NHIF did not cover included dental services, eye examination, physiotherapy, x-ray and laboratory tests such as glycated haemoglobin test (HbA1c), liver or kidney function tests, urea, electrolyte, creatinine (UECs), among others.

"… when you've been sent to physiotherapy. They will tell you to add a certain amount of money that they will tell you. They say that the card does not cover that." Urban FGD Participant, County A

"Some tests like for the diabetics, if you want to do an HbA1c, they have to pay cash. If they have to do a thyroid function test, they have to pay cash. So, there are some tests, even PSA [prostate-specific antigen]*, for the men with the NCD, they have to pay in cash. So, I have really never understood why, whereas when they go to NHIF they are told it is fully covered by NHIF."* Healthcare worker, County Referral Hospital, County B.

As a result of these additional costs, some patients were reported to skip clinic visits hence compromising continuity of care.

"First of all, he has issues with the fare to the facility. Reaching the facility, he has to be sent to go and do an RBS [random blood sugar] *or an FBS* [fasting blood sugar]*. That is an extra cost. From there he has been told to go buy drugs for 3 months and he is unable to even buy worth one week. So, he might end up absconding from clinic or just stopping the care completely."* County Official, County A

For peer review only - http://bmjopen.bmj.com/site/about/guidelines.xhtml

DISCUSSION

This study aimed to assess the responsiveness of the NHIF general scheme to the needs of individuals with hypertension and/or diabetes. Our findings are consistent with international evidence that having health insurance can enhance access to NCDs services [18-20]. Study respondents reported that having NHIF was critical in improving access to NCDs services. However, purchaser (NHIF) and provider (healthcare facilities) factors continue to impair the responsiveness of the NHIF to the needs of individuals living with NCDs. On the purchaser side, documented challenges included inadequate awareness about service entitlements in the NHIF Supa Cover package. Inadequate awareness of NCD service entitlements by healthcare workers and patients resulted in denial of care as patients would sometimes be turned away. A similar finding has been reported in a previous study [35]. In addition, respondents felt that the NHIF Supa Cover did not prioritise preventive and promotive services for NCDs but rather focused on curative services at secondary hospitals, that are not accessible to the rural poor. Nevertheless, targeted NCD screening is cost-effective[49-51] and there is evidence that the poor need NCD screening services more than the rich in Kenya [13].

Unaffordability of the NHIF Supa Cover monthly premiums and the penalties of defaulting payment were among the key weaknesses identified by study participants. This was specifically a challenge for enrolees from the informal sector, a finding that has been reported in previous studies conducted in Ghana and Kenya [35, 52, 53]. Furthermore, NCD patients enrolled in the NHIF Supa Cover from the informal sector were required to pay their monthly premium subscription at least one year in advance before NHIF provided authorisation for them to undergo costly or complex procedures. This presents a substantial financial barrier to patients and goes counter to the role of health insurance as a mechanism for pooling risks to avert the need to make large OOP payments when a health event occurs. Despite the NHIF providing some financial protection, OOP payments persisted. The unavailability of NCD medicines in public facilities has been corroborated by the findings of a previous study conducted in eight Kenyan counties.[54] Similarly, there is evidence of inequities in hypertension treatment in Kenya, with the poor being disproportionately disadvantaged [13].

Several purchaser and provider factors compromised physical access to NHIF-contracted facilities and quality of care for NCD services. First, except for inpatient admission, capitation-based payments by NHIF limited access to NCDs services to only one NHIF-accredited provider. As such, NCD patients had to pay OOP to access NCD services from other providers if the services they needed were unavailable from the providers where they were capitated. Second, infrastructural gaps hindered access and compromised the quality of NCDs services as patients were sometimes

For peer review only - http://bmjopen.bmj.com/site/about/guidelines.xhtml

turned away and the required laboratory supplies and equipment were often broken down or lacking in most public facilities contracted by NHIF.

Whereas this study did not formally assess provider factors that influence service experience by individuals enrolled in the NHIF Supa Cover, there were provider factors that influenced respondents' experience of the Supa Cover benefit package. For instance, consistent with available literature [29, 55], some NCD patients felt discriminated against by healthcare workers because they were enrolled in NHIF. Healthcare workers' negative attitudes towards NHIF enrolees is likely to lead to attrition [35]. This finding could be explained by NHIF's capitation-based provider payment mechanisms that are low and unpredictable [56].

This study had limitations. First, while we aimed to elicit experiences from NCD patients who were enrolled in the NHIF Supa Cover scheme at the time of the interview, including NCD patients who had discontinued membership from NHIF could have yielded richer findings on NHIF responsiveness. Second, the study was limited to two counties, and a larger study across more counties may identify a greater range of experiences. However, the sample included urban and rural sites and health staff from a range of cadres and provides evidence of NHIF Supa Cover gaps that need urgent attention to enhance its responsiveness to the needs of NCD patients.

Based on our study findings, we make the following policy recommendations. First, the NHIF should enhance its engagement and communication with its members living with NCDs, health facilities and county managers to improve awareness about the entitlements in the NHIF benefit package. Second, the NHIF should review its benefit package to align with the needs of people living with NCDs. This includes considering the inclusion of key diagnostic, and monitoring services for NCDs, and expanding priority to include preventive and promotive services which are more cost-effective. Third, the NHIF should review the design of the outpatient capitation model to make provisions for portability. That is, the capitation mechanism should provide for individuals to access services in facilities that they are not capitated to in instances when this is necessary (such as when they travel or in case of an emergency). Fourth, the NHIF and county governments should strengthen monitoring and accountability of health facilities to address undesired provider behaviour such as discrimination. Fifth, to address the structural quality issues of health facilities, the NHIF and county governments should enhance monitoring. Further, the NHIF should enhance its facility selection mechanism to assure the selection of health facilities with the structural capacity to deliver high-quality services, while the county government and health facilities should prioritise investments to improve the structural capacity of public health facilities. Health facility managers also have a role in implementing internal mechanism to prevent and address undesired

For peer review only - http://bmjopen.bmj.com/site/about/guidelines.xhtml

provider behaviour through monitoring and sanctions. Sixth, the national government should consider providing full or partial subsidies to enhance the affordability of the NHIF premiums in the informal sector. This should be accompanied by a review of the additional administrative charges to enrol with the NHIF. Lastly, the NHIF should review its pre-authorisation requirements to remove financial barriers. Specifically, the requirement for NHIF members from the informal sector to make long-term premium payments before accessing expensive procedures goes counter to the intention to provide financial risk protection through pooling.

Conclusion

While the NHIF has reduced financial barriers and enhanced access to care for people living with hypertension and diabetes in Kenya, challenges persist that compromise NHIF's responsiveness to the needs of these patients. As the government of Kenya embarks on the national roll out its national UHC programme, a window of opportunity exists to enhance NHIF's responsiveness to the needs of NCD patients. The responsibility to do this is shared among key actors like the NHIF, the national and county governments, as well as health facilities.

Acknowledgment: The authors wish to acknowledge the county health officials from Busia and Trans Nzoia, research assistants and study participants for enabling the successful implementation of this research study

Contributions: PP, JHK, EN, VN, AM, AE and EB conceptualised the study and obtained funding. EB, VW, RO, RW and HK contributed to the methodological development and study design. HK, RM, RO, and VW supported participant recruitment and data collection. EB, RW and RO analysed and interpreted the data. RO prepared the first draft manuscript that was subsequently reviewed and revised by all the authors. All authors read and approved the final manuscript.

Funding: This work was supported by the UK Medical Research Council grant number MR/T023538/1. RO is funded by a Wellcome Trust International Masters award (#214622), and additional funds from a Wellcome Trust core grant awarded to the KEMRI-Wellcome Trust Research Program (#092654) supported this work.

Competing interests: None declared

Data availability statement: No additional data available

For peer review only - http://bmjopen.bmj.com/site/about/guidelines.xhtml

For peer review only

REFERENCES

1. Naghavi, M., et al., *Global, regional, and national age-sex specific mortality for 264 causes of death, 1980–2016: a systematic analysis for the Global Burden of Disease Study 2016.* The Lancet, 2017. **390**(10100): p. 1151-1210.
2. World Health Organization, *Global status report on noncommunicable diseases 2014*. 2014, World Health Organization.
3. World Health Organization, *Noncommunicable diseases country profiles 2018.* . 2018: Geneva: World Health Organization; 2018. Licence: CC BY-NC-SA 3.0 IGO.
4. Gouda, H.N., et al., *Burden of non-communicable diseases in sub-Saharan Africa, 1990–2017: results from the Global Burden of Disease Study 2017.* The Lancet Global Health, 2019. **7**(10): p. e1375-e1387.
5. Vos, T., et al., *Global burden of 369 diseases and injuries in 204 countries and territories, 1990–2019: a systematic analysis for the Global Burden of Disease Study 2019.* The Lancet, 2020. **396**(10258): p. 1204-1222.
6. Watkins, D.A., et al., *Universal health coverage and essential packages of care.* 2018.
7. World Health Organization, *Health systems financing: the path to universal coverage. Geneva: World Health Organization.* 2012.
8. Ataklte, F., et al., *Burden of undiagnosed hypertension in sub-saharan Africa: a systematic review and meta-analysis.* Hypertension, 2015. **65**(2): p. 291-298.
9. International Diabetes Federation, *IDF Diabetes Atlas, 9th edn.* 2019: Brussels, Belgium: International Diabetes Federation.
10. Murphy, A., et al., *Inequalities in the use of secondary prevention of cardiovascular disease by socioeconomic status: evidence from the PURE observational study.* The Lancet Global Health, 2018. **6**(3): p. e292-e301.
11. Sozmen, K. and U. Belgin, *Socioeconomic inequalities in non-communicable diseases and self assessed health in Turkey.* Iranian journal of public health, 2014. **43**(6): p. 736.
12. Vellakkal, S., et al., *Are estimates of socioeconomic inequalities in chronic disease artefactually narrowed by self-reported measures of prevalence in low-income and middle-income countries? Findings from the WHO-SAGE survey.* J Epidemiol Community Health, 2015. **69**(3): p. 218-225.
13. Oyando, R., E. Barasa, and J.E. Ataguba, *Socioeconomic Inequity in the Screening and Treatment of Hypertension in Kenya: Evidence From a National Survey.* Frontiers in Health Services, 2022: p. 7.
14. Nugent, R., et al., *Investing in non-communicable disease prevention and management to advance the Sustainable Development Goals.* The Lancet, 2018. **391**(10134): p. 2029-2035.
15. United Nations, *Transforming Our World - the 2030 Agenda for Sustainable Development.* *https://sustainabledevelopment.un.org/topics/sustainabledevelopmentgoals (Accessed 18 February 2022).* 2015.
16. Jamison, D.T., et al., *Global health 2035: a world converging within a generation.* The lancet, 2013. **382**(9908): p. 1898-1955.
17. Kazibwe, J., P.B. Tran, and K.S. Annerstedt, *The household financial burden of non-communicable diseases in low-and middle-income countries: a systematic review.* Health research policy and systems, 2021. **19**(1): p. 1-15.
18. El-Sayed, A.M., et al., *Does health insurance mitigate inequities in non-communicable disease treatment? Evidence from 48 low-and middle-income countries.* Health Policy, 2015. **119**(9): p. 1164-1175.
19. Wagner, A.K., et al., *Access to care and medicines, burden of health care expenditures, and risk protection: results from the World Health Survey.* Health policy, 2011. **100**(2-3): p. 151-158.
20. Fang, J., et al., *Insurance status among adults with hypertension—the impact of underinsurance.* Journal of the American Heart Association, 2016. **5**(12): p. e004313.

For peer review only - http://bmjopen.bmj.com/site/about/guidelines.xhtml

21. Barasa, E., et al., *Examining the level and inequality in health insurance coverage in 36 sub-Saharan African countries.* BMJ Global Health, 2021. **6**(4): p. e004712.
22. Ly, M.S., O. Bassoum, and A. Faye, *Universal health insurance in Africa: a narrative review of the literature on institutional models.* BMJ Global Health, 2022. **7**(4): p. e008219.
23. Ministry of Health, *Kenya STEPwise survey for non-communicable diseases risk factors 2015 report. Available at:* http://aphrc.org/wp-content/uploads/2016/04/Steps-Report-NCD-2015.pdf *[Accessed 8 February 2022]*. 2015.
24. Barasa, E.W., T. Maina, and N.J.I.j.f.e.i.h. Ravishankar, *Assessing the impoverishing effects, and factors associated with the incidence of catastrophic health care payments in Kenya.* 2017. **16**(1): p. 1-14.
25. Oyando, R., et al., *Patient costs of hypertension care in public health care facilities in Kenya.* The International journal of health planning and management, 2019. **34**(2): p. e1166-e1178.
26. Oyando, R., et al., *Patient costs of diabetes mellitus care in public health care facilities in Kenya.* The International Journal of Health Planning and Management, 2020. **35**(1): p. 290-308.
27. Subramanian, S., et al., *Cost and affordability of non-communicable disease screening, diagnosis and treatment in Kenya: Patient payments in the private and public sectors.* PloS one, 2018. **13**(1): p. e0190113.
28. Barasa, E., et al., *Kenya National Hospital Insurance Fund Reforms: implications and lessons for universal health coverage.* Health Systems & Reform, 2018. **4**(4): p. 346-361.
29. Kabia, E., et al., *"We are called the et cetera": experiences of the poor with health financing reforms that target them in Kenya.* International Journal for Equity in Health, 2019. **18**(1): p. 98.
30. Tama, E., et al., *Examining the implementation of the free maternity services policy in Kenya: a mixed methods process evaluation.* International journal of health policy and management, 2018. **7**(7): p. 603.
31. Chuma, J., et al., *Reducing user fees for primary health care in Kenya: Policy on paper or policy in practice?* International journal for equity in health, 2009. **8**(1): p. 1-10.
32. Munge, K., et al., *A critical analysis of purchasing arrangements in Kenya: the case of the National Hospital Insurance Fund.* International journal of health policy and management, 2018. **7**(3): p. 244.
33. Kazungu, J.S. and E.W. Barasa, *Examining levels, distribution and correlates of health insurance coverage in Kenya.* Tropical Medicine & International Health, 2017. **22**(9): p. 1175-1185.
34. Mbau, R., et al., *Examining purchasing reforms towards universal health coverage by the National Hospital Insurance Fund in Kenya.* International journal for equity in health, 2020. **19**(1): p. 1-18.
35. Barasa, E.W., et al., *Extending voluntary health insurance to the informal sector: experiences and expectations of the informal sector in Kenya.* Wellcome open research, 2017. **2**.
36. Nolte, E., et al., *Scaling up the primary health integrated care project for chronic conditions in Kenya: study protocol for an implementation research project.* 2021.
37. World Bank, *The World Bank Kenya Profile:* http://data.worldbank.org/country/kenya *(Accessed February 17, 2022)*. 2022.
38. Government of Kenya, *The Constitution of Kenya*. 2010: Nairobi: Government of Kenya.
39. Ministry of Health, *Kenya Health Policy 2014-2030*. 2014.
40. Government of Kenya, *Kenya Service Availablity and Readiness Assessment Mapping (SARAM) Report.* 2013, Ministry of Health: Nairobi, Kenya
41. Ministry of Health Kenya, *Kenya Health Sector Strategic and Investment Plan: Transforming Health: Accelerating Attainment of Universal Health Coverage.* 2014, Ministry of Health: Nairobi, Kenya.
42. Barasa, E., P. Nguhiu, and D. McIntyre, *Measuring progress towards sustainable development goal 3.8 on universal health coverage in Kenya.* BMJ global health, 2018. **3**(3): p. e000904.

For peer review only - http://bmjopen.bmj.com/site/about/guidelines.xhtml

43. Munge, K. and A.H. Briggs, *The progressivity of health-care financing in Kenya.* Health policy and planning, 2013. **29**(7): p. 912-920.
44. Ministry of Health, *Republic of Kenya. National Strategic Plan for the Prevention and Control of Non-Communicable Diseases 2021/22–2025/26. Available* https://www.health.go.ke/wp-content/uploads/2021/07/Kenya-Non-Communicable-Disease-NCD-Strategic-Plan-2021-2025.pdf *(Accessed 24 June 2022)*. 2021: Nairobi, Kenya
45. Manji, I., et al., *The revolving fund pharmacy model: backing up the ministry of health supply chain in western Kenya.* International Journal of Pharmacy Practice, 2016. **24**(5): p. 358-366.
46. KNBS, *Kenya population and housing census*. 2019.
47. Ministry of Health. *Kenya Master Health Facility List, 2020:* https://open.africa/dataset/kenya-master-health-facility-list-2020 *(Accessed 01 March 2022)*. 2020.
48. Braun, V. and V. Clarke, *Using thematic analysis in psychology.* Qualitative research in psychology, 2006. **3**(2): p. 77-101.
49. Howard, K., et al., *Cost-effectiveness of screening and optimal management for diabetes, hypertension, and chronic kidney disease: a modeled analysis.* Value in health, 2010. **13**(2): p. 196-208.
50. Hoerger, T.J., et al., *Screening for type 2 diabetes mellitus: a cost-effectiveness analysis.* Annals of Internal Medicine, 2004. **140**(9): p. 689-699.
51. Kahn, R., et al., *Age at initiation and frequency of screening to detect type 2 diabetes: a cost-effectiveness analysis.* The Lancet, 2010. **375**(9723): p. 1365-1374.
52. Agyepong, I.A., et al., *The "Universal" in UHC and Ghana's National Health Insurance Scheme: policy and implementation challenges and dilemmas of a lower middle income country.* BMC Health Services Research, 2016. **16**(1): p. 1-14.
53. Okungu, V.R. and D. McIntyre, *Does the informal sector in Kenya have financial potential to sustainably prepay for health care? Implications for financing universal health coverage in low-income settings.* Health Systems & Reform, 2019. **5**(2): p. 145-157.
54. Ashigbie, P.G., et al., *Availability and prices of medicines for non-communicable diseases at health facilities and retail drug outlets in Kenya: a cross-sectional survey in eight counties.* BMJ open, 2020. **10**(5): p. e035132.
55. Jehu-Appiah, C., et al., *Household perceptions and their implications for enrolment in the National Health Insurance Scheme in Ghana.* Health policy and planning, 2012. **27**(3): p. 222-233.
56. Obadha, M., et al., *Health care purchasing in Kenya: Experiences of health care providers with capitation and fee-for-service provider payment mechanisms.* The International Journal of Health Planning and Management, 2019. **34**(1): p. e917-e933.

For peer review only

For peer review only - http://bmjopen.bmj.com/site/about/guidelines.xhtml

Figure 1: Study conceptual framework

For peer review only

For peer review only - http://bmjopen.bmj.com/site/about/guidelines.xhtml

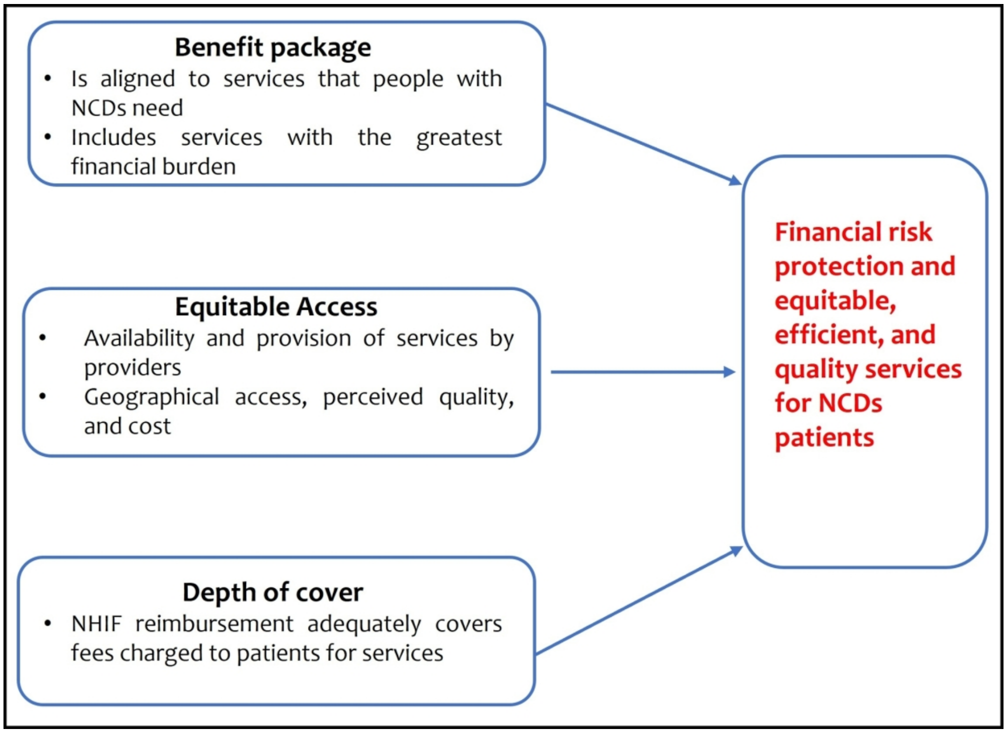

Figure 1 Study conceptual framework

173x125mm (300 x 300 DPI)

STROBE Statement—Checklist of items that should be included in reports of ***cross-sectional studies***

	Item No	Recommendation	Page No
**Title and abstract**	1	(*a*) Indicate the study's design with a commonly used term in the title or the abstract	1
		(*b*) Provide in the abstract an informative and balanced summary of what was done and what was found	2
**Introduction**			
Background/rationale	2	Explain the scientific background and rationale for the investigation being reported	3 to 5
Objectives	3	State specific objectives, including any prespecified hypotheses	
**Methods**			
Study design	4	Present key elements of study design early in the paper	7
Setting	5	Describe the setting, locations, and relevant dates, including periods of recruitment, exposure, follow-up, and data collection	6
Participants	6	(*a*) Give the eligibility criteria, and the sources and methods of selection of participants	7
Variables	7	Clearly define all outcomes, exposures, predictors, potential confounders, and effect modifiers. Give diagnostic criteria, if applicable	NA
Data sources/ measurement	8*	For each variable of interest, give sources of data and details of methods of assessment (measurement). Describe comparability of assessment methods if there is more than one group	NA
Bias	9	Describe any efforts to address potential sources of bias	8
Study size	10	Explain how the study size was arrived at	7 to 8
Quantitative variables	11	Explain how quantitative variables were handled in the analyses. If applicable, describe which groupings were chosen and why	NA
Statistical methods	12	(*a*) Describe all statistical methods, including those used to control for confounding	NA
		(*b*) Describe any methods used to examine subgroups and interactions	NA
		(*c*) Explain how missing data were addressed	NA
		(*d*) If applicable, describe analytical methods taking account of sampling strategy	NA
		(*e*) Describe any sensitivity analyses	NA
**Results**			
Participants	13*	(a) Report numbers of individuals at each stage of study—eg numbers potentially eligible, examined for eligibility, confirmed eligible, included in the study, completing follow-up, and analysed	10
		(b) Give reasons for non-participation at each stage	NA
		(c) Consider use of a flow diagram	NA
Descriptive data	14*	(a) Give characteristics of study participants (eg demographic, clinical, social) and information on exposures and potential confounders	10
		(b) Indicate number of participants with missing data for each variable of interest	NA
Outcome data	15*	Report numbers of outcome events or summary measures	NA

For peer review only

Main results	16	(*a*) Give unadjusted estimates and, if applicable, confounder-adjusted estimates and their precision (eg, 95% confidence interval). Make clear which confounders were adjusted for and why they were included	NA
		(*b*) Report category boundaries when continuous variables were categorized	NA
		(*c*) If relevant, consider translating estimates of relative risk into absolute risk for a meaningful time period	NA
Other analyses	17	Report other analyses done—eg analyses of subgroups and interactions, and sensitivity analyses	NA
**Discussion**			
Key results	18	Summarise key results with reference to study objectives	16
Limitations	19	Discuss limitations of the study, taking into account sources of potential bias or imprecision. Discuss both direction and magnitude of any potential bias	17
Interpretation	20	Give a cautious overall interpretation of results considering objectives, limitations, multiplicity of analyses, results from similar studies, and other relevant evidence	17
Generalisability	21	Discuss the generalisability (external validity) of the study results	17
**Other information**			
Funding	22	Give the source of funding and the role of the funders for the present study and, if applicable, for the original study on which the present article is based	18

*Give information separately for exposed and unexposed groups.

Note: An Explanation and Elaboration article discusses each checklist item and gives methodological background and published examples of transparent reporting. The STROBE checklist is best used in conjunction with this article (freely available on the Web sites of PLoS Medicine at http://www.plosmedicine.org/, Annals of Internal Medicine at http://www.annals.org/, and Epidemiology at http://www.epidem.com/). Information on the STROBE Initiative is available at www.strobe-statement.org.

For peer review only

For peer review only - http://bmjopen.bmj.com/site/about/guidelines.xhtml

BMJ Open

Examining the Responsiveness of the National Health Insurance Fund (NHIF) to People Living with Hypertension and Diabetes in Kenya: A qualitative study

Journal:	*BMJ Open*
Manuscript ID	bmjopen-2022-069330.R1
Article Type:	Original research
Date Submitted by the Author:	20-Feb-2023
Complete List of Authors:	Oyando, Robinson; KEMRI-Wellcome Trust Research Programme<br>Were, Vincent; KEMRI-Wellcome Trust Research Programme Nairobi, Health Economics Research Unit (HERU)<br>Willis, Ruth; London School of Hygiene and Tropical Medicine Faculty of Public Health and Policy, Department of Health Service Research and Policy<br>Koros, Hillary; Academic Model Providing Access to Healthcare<br>Kamano, Jemima; Moi University, Department of Medicine, School of Medicine, College of Health Sciences,<br>Naanyu, Violet; Moi University, School of Arts and Social Sciences; Moi University College of Health Sciences, Department of Medicine<br>Etyang, Anthony ; KEMRI-Wellcome Trust Research Programme, Department of Epidemiology and Demography<br>Mugo, Richard; AMPATH Kenya<br>Murphy, Adrianna; London School of Hygiene and Tropical Medicine Faculty of Public Health and Policy,<br>Nolte, Ellen; London School of Hygiene & Tropical Medicine, Department of Health Services Research and Policy<br>Perel, Pablo; LSMTM<br>Barasa, Edwine; Centre for Geographic Medicine Research Coast, Health Economics Research Unit
**Primary Subject Heading**:	Health economics
Secondary Subject Heading:	Qualitative research, Health economics, Public health, Health policy, Health services research
Keywords:	Hypertension < CARDIOLOGY, Health policy < HEALTH SERVICES ADMINISTRATION & MANAGEMENT, Health economics < HEALTH SERVICES ADMINISTRATION & MANAGEMENT, QUALITATIVE RESEARCH, PUBLIC HEALTH

SCHOLARONE™
Manuscripts

I, the Submitting Author has the right to grant and does grant on behalf of all authors of the Work (as defined in the below author licence), an exclusive licence and/or a non-exclusive licence for contributions from authors who are: i) UK Crown employees; ii) where BMJ has agreed a CC-BY licence shall apply, and/or iii) in accordance with the terms applicable for US Federal Government officers or employees acting as part of their official duties; on a worldwide, perpetual, irrevocable, royalty-free basis to BMJ Publishing Group Ltd ("BMJ") its licensees and where the relevant Journal is co-owned by BMJ to the co-owners of the Journal, to publish the Work in this journal and any other BMJ products and to exploit all rights, as set out in our licence.

The Submitting Author accepts and understands that any supply made under these terms is made by BMJ to the Submitting Author unless you are acting as an employee on behalf of your employer or a postgraduate student of an affiliated institution which is paying any applicable article publishing charge ("APC") for Open Access articles. Where the Submitting Author wishes to make the Work available on an Open Access basis (and intends to pay the relevant APC), the terms of reuse of such Open Access shall be governed by a Creative Commons licence – details of these licences and which Creative Commons licence will apply to this Work are set out in our licence referred to above.

Other than as permitted in any relevant BMJ Author's Self Archiving Policies, I confirm this Work has not been accepted for publication elsewhere, is not being considered for publication elsewhere and does not duplicate material already published. I confirm all authors consent to publication of this Work and authorise the granting of this licence.

For peer review only

Examining the Responsiveness of the National Health Insurance Fund (NHIF) to People Living with Hypertension and Diabetes in Kenya: A qualitative study

Robinson Oyando[1], Vincent Were[1], Ruth Willis[2], Hillary Koros[3], Jemima Kamano[4], Violet Naanyu[4,5], Anthony Etyang[6], Richard Mugo[3], Adrianna Murphy[2], Ellen Nolte[2], Pablo Perel[7], Edwine Barasa[1,8]

[1] Health Economics Research Unit, KEMRI-Wellcome Trust Research Programme, Nairobi, Kenya
[2] Department of Health Services Research and Policy, London School of Hygiene & Tropical Medicine, London, UK
[3]AMPATH, Eldoret, Kenya
[4] Department of Medicine, School of Medicine, College of Health Sciences, Moi University, Eldoret, Kenya
[5] School of Arts and Social Sciences, Moi University, Eldoret, Kenya
[6] Department of Epidemiology and Demography, KEMRI-Wellcome Trust Research Programme, Kilifi, Kenya
[7] Department of Non-Communicable Disease Epidemiology, London School of Hygiene and Tropical Medicine, London
[8] Center for Tropical Medicine and Global Health, Oxford University, Oxford, 01540, UK

Corresponding author:

Robinson Oyando
Health Economics Research Unit, KEMRI Wellcome Trust Research Programme,
P.O.BOX 43640-00100,
Nairobi, Kenya
Email: ROyando@kemri-wellcome.org

For peer review only

For peer review only - http://bmjopen.bmj.com/site/about/guidelines.xhtml

ABSTRACT

Objectives: To assess the responsiveness of the National Health Insurance Fund (NHIF) Supa Cover benefit package to the needs of individuals with diabetes and hypertension in Kenya.

Design, setting and participants: We carried out a qualitative study and collected data using key informant interviews (n = 39) and focus group discussions (n = 4) in two purposively selected counties in Western Kenya. Study participants were drawn from NHIF officials, county government officials, health facility managers, healthcare workers and individuals with hypertension and diabetes who were enrolled in NHIF. We analysed data using a thematic approach.

Results: Study participants reported that the NHIF Supa Cover benefit package expanded access to services for people living with hypertension and diabetes. However, the NHIF members and healthcare workers had inadequate awareness of the NHIF service entitlements. The NHIF benefit package inadequately covered the range of services needed by people living with hypertension and diabetes and the benefits package did not prioritise preventive and promotive services. Sometimes patients were discriminated against by healthcare providers who preferred cash-paying patients, and some NHIF-empanelled health facilities had inadequate structural inputs essential for quality of care. Study participants felt that the NHIF premium for the general scheme was unaffordable, and NHIF members faced additional out-of-pocket costs because of additional payments for services not available or covered.

Conclusion: Whereas NHIF has reduced financial barriers for hypertension and diabetes patients, to enhance its responsiveness to patient needs, NHIF should implement mechanisms to increase benefit package awareness among members and providers. In addition, preventive and promotive services should be included in NHIF's benefits package and mechanisms to monitor and hold contracted providers accountable should be strengthened.

Keywords: Universal health coverage, non-communicable diseases, reforms, Kenya, Low- and Middle-Income Countries

For peer review only - http://bmjopen.bmj.com/site/about/guidelines.xhtml

Strengths and limitations of this study

- This study used in-depth interviews and focus group discussions to comprehensively examine how responsive the national health insurance fund is to the healthcare needs of patients with hypertension and diabetes.
- The study recruited participants from both urban and rural sites and health staff from a range of cadres and thus provides evidence from a wide range of respondents with varied experiences.
- Including hypertension and diabetes patients who had discontinued membership from the national health insurance scheme could have yielded richer findings on the scheme's responsiveness.

INTRODUCTION

Non-communicable diseases (NCDs) account for the greatest share of the global disease burden and are one of the significant health challenges of the century [1, 2]. Low-and-middle income countries (LMICs) bear the highest NCD burden [3]. While infectious diseases contribute to the highest share of disease burden in sub-Saharan Africa (SSA), the burden of NCDs is rapidly increasing and is expected to grow [4]. In Kenya, NCDs account for 50% of inpatient admissions and 55% of hospital deaths [5]. Responsive health systems provide high-quality health services equitably and efficiently to the population in need without the risk of catastrophic or impoverishing healthcare expenditures [6, 7]. Evidence suggests that most NCD patients in LMICs do not have equal access to NCD services such as screening and treatment [8-13]. Among other things, this is due to lack of access due to financial barriers and inadequate capacity of health facilities to offer NCD care [8, 12-14].

One of the mechanisms to enable financial access to NCD services is designing and implementing pre-payment health financing mechanisms from tax or compulsory insurance contributions that provide financial risk protection to households and individuals living with NCDs and enhance access

For peer review only - http://bmjopen.bmj.com/site/about/guidelines.xhtml

to quality NCD services [15, 16]. Some evidence suggests that having health insurance is beneficial in enabling access to NCDs services [17-19]. Nevertheless, health insurance coverage in SSA remains low, with only four out of 36 SSA countries having health insurance coverage of any type exceeding 20% [20]. In addition, tax-based pre-payment mechanisms are preferred as an equitable health financing mechanism in SSA given the context of a large informal sector with a low contributory capacity [21].

Kenya has implemented several health financing initiatives [22-25]. One such initiative is the policy decision to use the National Health Insurance Fund (NHIF) as one of the key pre-payment health financing mechanisms [26]. NHIF is a public insurer in Kenya that was established in 1966 to provide mandatory health insurance to all Kenyans [22]. However, national NHIF coverage remains low, at 24% [27]. In the past 11 years, several reforms have been introduced in NHIF in an effort to enhance its capacity to deliver UHC [22]. Among these reforms is the broadening of the benefit package ('Supa Cover') of its general scheme (that targets all Kenyans) to include outpatient and specialised services for NCDs [22].

There is limited evidence about the degree to which the expanded NHIF national scheme is addressing the needs of people with hypertension and diabetes—two major NCDs in Kenya. This study aims to generate evidence that would be relevant in informing policymakers and other stakeholders on how the configuration of health insurance reforms can be tailored to address the needs of NCD patients and thus contribute to the achievement of international benchmarks for NCDs spelt out in Sustainable Development Goal 3.4.

METHODS

Study setting

Kenya is a lower-middle income (LMIC) country located in the East Africa region [28]. There are two levels of administration in Kenya's health system: national and county [29]. The national government (Ministry of Health) is responsible for policy and regulation in the health system while the 47 county governments are responsible for service delivery [30]. The Kenya health system is pluralistic, with an almost equal share of service provision by the public and private providers [31].

Kenya's health system has a four-tiered structure. Tier 1 is at the community level where community-based healthcare demand creation activities are undertaken as stipulated in the community health strategy; Tier 2 is at the primary care level where preventive and promotive services are provided in dispensaries and health centres; Tier 3 consists of both primary and

For peer review only - http://bmjopen.bmj.com/site/about/guidelines.xhtml

secondary referral hospitals; and Tier 4 consists of tertiary referral hospitals under the direct management of the Ministry of Health (MoH) [32].

The sources of health financing in Kenya include out-of-pocket (OOP) payments by households, government (national and county) tax revenues, donors, and member contributions from NHIF and private insurance companies [33, 34]. For instance, in the financial year (FY) 2015/16, current health expenditure (CHE) as a percentage of gross domestic product was 5.2%, CHE per capita in US$ was 78.6 while the proportion of CHE financed through OOP payments was 26.1% [33]. In addition, the total health expenditure (THE) on NCDs as a proportion of THE was 11% in FY 2017/18 [35].

For peer review only

For peer review only - http://bmjopen.bmj.com/site/about/guidelines.xhtml

Study sites

This study was carried out in Busia and Trans Nzoia counties in Western Kenya, where Moi University, Moi Teaching and Referral Hospital through AMPATH (Academic Model Providing Access to Healthcare) have partnered for several years with county governments to strengthen health systems across various care levels [36].

This study is part of a larger study that seeks to inform and support the scale-up of the Primary Health Integrated Care Project for Chronic Conditions (PIC4C) model for integrated management of people with hypertension, diabetes, and breast and cervical cancers in Kenya [37]. Alongside other work packages in the larger study, our work package had three overarching objectives: 1) to measure the effectiveness of the NHIF national scheme benefit package to provide financial risk protection to individuals with hypertension/diabetes; 2) to examine the extent to which the NHIF national scheme benefit package is responsive to the needs of individuals with hypertension/diabetes; and 3) to examine how the provider incentives generated by provider payment arrangements of the NHIF national scheme benefit package influence equity, efficiency, and quality of care. Our engagement with policy makers on the findings of the entire study included presentation of findings from both the quantitative (objective 1) and qualitative (objectives 2 and 3) work. However, the focus of this paper is on objective 2.

Table 1 outlines the key characteristics of the two counties. Launched in 2018, the PIC4C model was implemented by a partnership between AMPATH/Moi, the MoH and the World Bank and included (i) early case finding of people with hypertension, diabetes, cervical/ breast cancer at service level 1; (ii) structured referral to service providers at level 2 for confirmation of diagnosis and treatment initiation or referral to level 3 or 4 using structured protocols; (iii) initiation of treatment using structured treatment protocols and decision support tools at levels 2, 3 and 4; (iv) retention of patients in care supported by ongoing training of health workers at all care levels; (v) monitoring and evaluation supported by a health information system; and (vi) advising patients in care to enrol in the NHIF national scheme for sustainable health financing [37].

For peer review only - http://bmjopen.bmj.com/site/about/guidelines.xhtml

Table 1 Characteristics of the study setting

Characteristics	Busia county	Trans Nzoia county
Population[ǂ]	893,681	990,341
% Adults (18+ years) [ǂ]	52%	53%
% Females [ǂ]	52%	51%
Life expectancy at birth[ǂǂ]	47 years	60.5 years
Poverty level[ǂǂ]	64%	50%
Estimated hypertension prevalence*	23.1%	33.6%
Estimated diabetes prevalence[ǂǂ]	1.5%	2%
NHIF coverage[ǂǂ]	31%	20%
Number of health facilities[†]	184 community units served by 47 dispensaries, 12 health centers, 5 sub-county and 1 county hospital	198 community units served by 38 dispensaries, 8 health centers, 6 sub-county health facilities and 1 county hospital
Number of patients with selected NCDs currently being serviced in AMPATH sites[ǂǂ]	6,060 patients with hypertension, 1,113 with diabetes and 200 with cervical cancer.	4,375 patients with hypertension, and 2,089 with diabetes.
Number of facilities where the PIC4C pilot was implemented[ǂǂ]	40 facilities (5 subcounty hospitals, 12 health centers and 23 dispensaries).	33 facilities (6 subcounty hospitals, 8 health centers and 19 dispensaries).

(Sources: ǂǂ[37];ǂ[38]; †[39];*[40])

Study conceptual framework

This study's conceptual framework (Figure 1) assumes that for the NHIF to be effective in providing financial risk protection to hypertension/diabetes patients and their households while enhancing access to quality health services, it is imperative that 1) the benefit package represents services that patients need 2) that patients actually access the entitlements from the benefit package, and 3) the NHIF reimbursements to healthcare providers adequately covers the fees providers charge patients for services (depth of cover). Therefore, following the study's conceptual framework, responsiveness in this study is conceptualised to mean that the NHIF Supa Cover meets the needs of NCD patients in each of the three elements.

Insert Figure 1 here.

For peer review only - http://bmjopen.bmj.com/site/about/guidelines.xhtml

Study design and data collection

We conducted a qualitative study where we interviewed purposively selected participants. We conducted four focus group discussions (FGDs) with NCD patients in Busia (1 rural and 1 urban) and Trans Nzoia (1 rural and 1 urban) counties who were enrolled in the NHIF national scheme. FGD participants were selected from an existing PIC4-C scale-up study database (matched cohort wave 2) [37]. FGD participants were eligible if 1) they were actively enrolled in NHIF at the time of recruitment, 2) they had either diabetes or hypertension or comorbid (both diabetes and hypertension), 3) were more than 18 years of age, and 4) if they consented to participate in the study. Patients were purposively selected to ensure a range of age, gender, condition/s, and attendance at urban/rural facility. Eligible participants were invited to the FGD sessions by telephone. FGD sessions took place at the nearest public health facility and at a time convenient to the participants. In-depth interviews (IDI) participants were purposively selected to include a range of cadres in both counties. IDIs were held with county health officials (n = 7), county NHIF branch officers (n = 3), health facility managers (n = 12), and front-line healthcare workers (n = 17) composed of medical officers, pharmacists, nursing officers and clinical officers drawn from public providers (health centres [n=2], sub-county hospitals [n=3] and county hospital [n=1]) and private providers (faith-based facility [n=1] and health centre [n=1]). All study participants were consented and signed informed consent forms before participating in the interview sessions. FGD and IDI sessions were facilitated by two trained qualitative researchers. The FGDs were facilitated in Kiswahili while the IDIs were conducted in English. With consent from the participants, the sessions were audio recorded and complimentary notes were taken. FGDs lasted for an average of 48 minutes while the interviews lasted for an average of 35 minutes. Table 2 outlines the number of respondents interviewed by their respective categories.

For peer review only

For peer review only - http://bmjopen.bmj.com/site/about/guidelines.xhtml

Table 2 Summary of Respondents

County	Respondent	No. interviewed
Busia	County officials	4
	NHIF branch officers	1
	Facility managers	6
	Frontline healthcare workers	10
	NCD patient FGDs	2 (1 urban [7 participants]; 1 rural [6 participants])
Trans Nzoia	County officials	3
	NHIF branch officers	2
	Facility managers	6
	Frontline healthcare workers	7
	NCD patient FGDs	2 (1 urban [7 participants]; 1 rural [5 participants])

The FGDs and IDIs were conducted using semi-structured topic guides. The development of the questions in the discussion and interview guides was informed by the study's conceptual framework (Figure 1). The questions focused on hypertension/diabetes services that NHIF pays for, adequacy of the range of services covered by NHIF, participants' experience with service provision, quality of care, prioritisation of preventive and promotive services in the NHIF Supa cover scheme, NHIF provider payment mechanisms, strengths, and weaknesses of the NHIF national scheme. At the end of each session, the facilitator and note taker held debrief sessions which facilitated improving the interviewing process and revision of some questions to enhance clarity and inclusion of emerging themes that needed further exploration. Data collection was discontinued when data saturation was attained (i.e., when no new information was forthcoming). The total sample size (39 interviews and 4 FGDs) meets the recommendation for sample size (9-17 for interviews and 4-8 for FGDs) for qualitative studies to achieve saturation [41]. Data were collected between June and July 2021.

Data management and analysis

Qualitative FGDs and IDIs audio recordings were transcribed verbatim and translated to English where necessary. To validate and familiarise ourselves with the transcribed transcripts, we read and re-read the transcripts and ensured correctness by re-listening to sections of the audio recordings. Validated transcripts were imported into NVivo QSR 12 for coding and analysis. We used a thematic approach to analyse the qualitative data. Thematic analysis involved familiarisation with

For peer review only - http://bmjopen.bmj.com/site/about/guidelines.xhtml

the data through reading and re-reading the transcripts to identify emerging ideas, meanings, patterns and potential themes [42]. In the first stage of analysis, two authors (RO and RW) working independently coded the first few transcripts to identify key themes and developed the initial coding framework. This involved the generation of initial codes, grouping developed codes into descriptive themes by identifying matching patterns. Thereafter, a discussion among three authors (RO, RW and EB) was held where individual codes were compared, and a final coding framework was arrived at by consensus. Whereas the coding of the transcript was guided by the study's conceptual framework, as data coding progressed iteratively, the coding framework was updated based on the emerging codes. In the next step, RO charted the data. During charting, emerging themes are identified through a process where coded data is reorganised. In this stage, RO summarised the ideas from each code with illustrative quotes from the data. EB checked and refined coding charts developed by RO. In the final phase, summaries from each thematic heading were critically interpreted and synthesised to identify key policy-relevant findings.

Patient and public involvement statement

The roll out of the PIC4C model in the two counties was accomplished by involving village chiefs and elders in the process. Patients and members of the public were not involved in the development of the proposal, or the design of the research presented here.

For peer review only

For peer review only - http://bmjopen.bmj.com/site/about/guidelines.xhtml

RESULTS

We conducted a total of 39 IDIs (17 men and 22 women) and four FGD sessions with 28 participants (15 women and 13 men). Six FGD participants had diabetes, 13 had hypertension and nine had both diabetes and hypertension. The results are presented under five key thematic areas: access to hypertension/diabetes services, awareness of hypertension/diabetes service entitlements in the NHIF Supa Cover scheme, adequacy of the range of hypertension/diabetes services covered by NHIF, provider payment mechanisms for hypertension/diabetes services and affordability of NHIF Supa Cover scheme. Summaries of key findings are provided under each thematic area.

Access to Hypertension/Diabetes Services

The NHIF benefit package expanded access to services for people living with hypertension/diabetes by reducing financial barriers. Several participants reported that the NHIF package had facilitated access to a range of hypertension/diabetes care services that they would otherwise have had to pay for OOP. Services that were reported to be covered included inpatient care and referral to NHIF accredited healthcare facilities, and medications. For example, one patient noted:

"When I have the NHIF, I have hope that I can go to the hospital even if I don't have any money and I can get medication compared to the one who does not have the NHIF." Rural FGD Participant, County A

One healthcare worker specifically related NHIF coverage to increased clinic attendance:

"The strength of NHIF super cover is that it has alleviated the suffering of these patients. There are patients who had stopped coming to the clinic because of financial challenges, but with the NHIF cover, we found that almost all our patients are always coming for the clinic… those who are covered are always coming for the clinic without fail." Healthcare worker, Sub-County Hospital, County A

Being enrolled in NHIF was reported to have beneficial implications for patients' wider financial situations, for example, avoiding the need to borrow money or sell assets to pay for health care services.

"It prevents you from borrowing and reduces stress for you and even respect comes. It prevents you from selling the farm, animals and any other thing." Rural FGD Participant, County B

Further, financial barriers were reduced for all population groups because the NHIF did not limit the enrolment of individuals on age or pre-existing NCD condition, unlike private health insurance.

For peer review only - http://bmjopen.bmj.com/site/about/guidelines.xhtml

Awareness of Hypertension/Diabetes service entitlements

Some NHIF members and healthcare workers had inadequate awareness of the NHIF service entitlements. While some study respondents showed awareness of the hypertension/diabetes care services that are covered by the NHIF Supa Cover scheme, others indicated that they were not adequately aware of service entitlements under the scheme.

"If you were to get a patient who needed radiotherapy or chemotherapy in my facility, we don't offer those services. I don't know if NHIF caters for that. I don't know to what extent… it does not cover some services such as chemotherapy or radiotherapy. I don't know if it caters for them, am not sure. I hope it does." Healthcare worker, Sub-County Hospital, County A

Inadequate awareness of hypertension/diabetes service entitlements in the Supa Cover scheme resulted in some patients being wrongfully turned away by healthcare workers.

"I asked if the NHIF card can be used to do x-ray and whoever was there told me that it cannot, you have to go outside the hospital. I didn't go and that is when I realised that the NHIF can do x-ray. That is when I realised that there are others who get services while others do not. The last time there was a nurse who came and asked on my behalf and she was told that it was working, and I got the services." Urban FGD Participant, County B

Adequacy of the range of hypertension/diabetes services

The NHIF benefit package inadequately covered the range of services needed by people living with hypertension and diabetes. While the cover catered for outpatient consultation and inpatient admissions, there was a strong perception among most NHIF members and healthcare workers that the NHIF Supa Cover package did not adequately cover the range of diagnostic, monitoring, and medicines that they needed and did not explicitly cover hypertension/diabetes medicines. Several participants reported incidents of relevant services having to be covered OOP by service users themselves.

"One day I came here and they sent me to go get an x-ray. When I got there, I was told that they cannot cover for that expense. "Go out". When I got out there, I asked and was told KES 5,200/= to get the x-ray. I was perplexed." Urban FGD, County A

"First thing urinalysis, HbA1c [glycated haemoglobin] *and UEC* [urea, electrolytes and creatinine]. *UEC will guide us whether to start with insulin oral or metformin alone. Then afterwards you need to follow up on this patient after three months you need HbA1c, but you find most of our clients mostly*

For peer review only - http://bmjopen.bmj.com/site/about/guidelines.xhtml

those relying on insurances, they're not able to pay so I can comfortably say they are not adequately covered using NHIF." Medical Officer, Private Hospital, County A

The NHIF benefit package did not prioritise preventive and promotive services for hypertension/diabetes. Some study respondents felt that the NHIF Supa Cover does not prioritise preventive and promotive services such as screening for NCDs but instead prioritises curative care after a patient is diagnosed with hypertension or diabetes. It was further felt that the NHIF prioritised secondary and admission care in hospitals rather than primary care services at lower-level facilities.

"The Supa Cover mostly deals with curative aspects of treatment. So, it doesn't deal with preventive aspects of treatment. So, it waits for a client to fall sick, then they can get services. There is no such thing as a preventive service, maybe going for annual check-up unless of course, you are covered under the civil servant type of cover where you have access to one check-up per year." Facility Manager, Sub-County Hospital, County A

"We were told that NHIF is able to cater for health checks, routine screening. And unfortunately, that one is not being done for everybody; it is not for the supa cover, it is for these other packages." County Official, County B

Provider payment mechanisms for hypertension/diabetes services

The design of the outpatient capitation payment mechanism reduced geographical access to services. The capitation payment mechanism required patients to be registered to a specific health facility for outpatient services. Some participants mentioned that this limited access to outpatient services when patients travelled far from their registered facility, and hence compromised continuity of care.

"The government should look into that… [so that] *if you have NHIF, you can receive treatment anywhere. It is not a must that one chooses a hospital to receive treatment."* Rural FGD Participant, County A

For peer review only - http://bmjopen.bmj.com/site/about/guidelines.xhtml

Quality of hypertension/diabetes care

NHIF patients were sometimes discriminated against by healthcare providers. Some study participants felt that cash-paying patients received more attention and care from healthcare workers than hypertension/diabetes patients enrolled in NHIF. For instance, respondents living with hypertension/diabetes observed that healthcare workers did not give them the support they required when in a health facility once they mentioned that they had NHIF cards.

"When you walk in the corridors and meet a doctor and you tell them you have NHIF card, they look at you differently. It is like people see you as desperate because you are mentioning that card. I think they prefer cash because if you say you have the card, they don't want to assist you." Urban FGD Participant, County B

"There are times when you waste time walking around the hospital with no help until you decide to use cash instead of wasting the whole day. Sometimes they have a negative attitude if you talk to them about the card." Urban FGD Participant, County B

However, other participants had dissenting views as they reported that there was no difference in how healthcare workers handled hypertension/diabetes patients enrolled in NHIF and those who are not.

"No favouritism is there because one is using NHIF and the other one is using cash." Rural FGD Participant, County B

Some patients related how they were sometimes denied the healthcare services they needed because they had an NHIF card. These patients were instead asked to seek services from private providers.

"I was just given Nifedipine alone for blood pressure. I had my fellow patient who was given all the drugs because she paid cash. She was given all drugs. I left there asking myself what the card had done. I was discharged and told to go and buy drugs. I was given only one drug." Urban FGD Participant, County B

Some NHIF-empanelled health facilities had an inadequate structural quality of care. This was characterised by overcrowding at the clinics, erratic supply of hypertension/diabetes medicines, diagnostic supplies and equipment. Some participants reported that they had to return on another clinic day or seek services in other (often private) health facilities and incur OOP.

For peer review only - http://bmjopen.bmj.com/site/about/guidelines.xhtml

"Again, after you've come for clinic, there is a schedule, and patients are many. You are told that time is up for receiving your patient book. Yet you got delayed at the laboratory. I have seen others being returned. Their book gets rejected." Urban FGD Participant, County A

"The challenge that is there, it is like there are some tests that they don't do. Like today I did ECG [electrocardiogram], to know the condition of my heart because the other day I came to the hospital. I told the doctor that I had pain that I could not understand. Then I was told to come and do ECG. The machine for ECG was spoilt and because I wanted the service, I had to go outside the hospital, and I paid." Urban FGD Participant, County B

Some patients and healthcare workers reported that public health facilities often did not provide inpatient diets that were suitable for patients with hypertension and diabetes. The meals they were served during inpatient admission in public facilities were contrary to the dietary advice they were given to manage their conditions.

"If they prepare uji (porridge), it has a lot of sugar. And you have been told not to take sugar. Who will prepare yours now? If they knew that, they would set apart a ward." Rural FGD Participant, County A

Affordability and Financial Risk Protection

Study respondents felt that the NHIF premiums for the general scheme were unaffordable. The KES 500 per month premium was deemed to be beyond reach by some respondents, especially in rural areas. As a result, people living with hypertension/diabetes could not keep up with the premium payments and regularly defaulted.

"The NHIF should reduce the premiums since the people in the rural areas see KES 500 as a lot of cash and fail to register or remain active." Rural FGD Participant, County B

Furthermore, individuals from the informal sector (whose membership was voluntary) who needed to undergo costly or complicated procedures were required to pay their monthly premium subscription at least one year in advance before NHIF authorised them to undergo any procedure. This was deemed unaffordable by many respondents.

"NHIF members from the informal sector are normally required to pay premiums at least more than 2 years in advance, depending on the cost of the procedure that the person is required to do. They are

For peer review only - http://bmjopen.bmj.com/site/about/guidelines.xhtml

required to pay premiums for 2 years or more before the preauthorisation can be approved." Healthcare worker, FBO, County A

"If you're a NHIF contributor and self-employed, you pay the KES 500 package every month. You cannot get the surgery done unless you get the pre-authorised form, and you should have paid premiums upfront for 1 year for the surgery to be approved. You will find that most of these patients are old, some of them are unemployed so while they can pay KES 500 per month, it becomes a very big issue to pay for a year or more." County Official, County A

NHIF members faced additional out-of-pocket costs because of administration charges, input supply shortages, balance billing, and payments for services not covered. Due to supply-side inefficiencies, some patients reported that they were required to make OOP to purchase consumables like gloves or photocopying fees for receipts.

"Everyone paying for NHIF is struggling. Like the other day on Wednesday. The day has come, we get there, KES 10/= for photocopy and KES 50/= for gloves." Rural FGD Participant, County B

Further, the reimbursements NHIF paid providers for inpatient and outpatient services were deemed insufficient to cover the cost of offering the services. Hence, patients had to make additional payments to bridge the gap in service delivery costs.

"Per day NHIF payment for inpatient is KES 1,500, while outpatient capitation is KES 1,200 so they can actually surpass. In a day the bill can actually go beyond that because we are looking at for example cost of insulin, the cost of the drugs they are getting… So, in as much as NHIF would pay the KES 1,200, now the patient has to top up, yes." Facility Manager, County Referral Hospital, County A

Services that NHIF did not cover included dental services, eye examination, physiotherapy, x-ray and laboratory tests such as glycated haemoglobin test (HbA1c), liver or kidney function tests, urea, electrolyte, creatinine (UECs), among others.

"… when you've been sent to physiotherapy. They will tell you to add a certain amount of money that they will tell you. They say that the card does not cover that." Urban FGD Participant, County A

"Some tests like for the diabetics, if you want to do an HbA1c, they have to pay cash. If they have to do a thyroid function test, they have to pay cash. So, there are some tests, even PSA [prostate-specific antigen], *for the men with the NCD, they have to pay in cash. So, I have really never understood why, whereas when they go to NHIF they are told it is fully covered by NHIF."* Healthcare worker, County Referral Hospital, County B.

For peer review only - http://bmjopen.bmj.com/site/about/guidelines.xhtml

As a result of these additional costs, some patients were reported to skip clinic visits hence compromising continuity of care.

"First of all, he has issues with the fare to the facility. Reaching the facility, he has to be sent to go and do a RBS [random blood sugar] *or a FBS* [fasting blood sugar]*. That is an extra cost. From there he has been told to go buy drugs for 3 months and he is unable to even buy worth one week. So, he might end up absconding from clinic or just stopping the care completely."* County Official, County A

For peer review only

For peer review only - http://bmjopen.bmj.com/site/about/guidelines.xhtml

DISCUSSION

This study aimed to assess the responsiveness of the NHIF general scheme to the needs of individuals with hypertension and/or diabetes. Our findings are consistent with international evidence that having health insurance can enhance access to NCDs services [17-19]. Study respondents reported that having NHIF was critical in improving access to NCDs services. However, purchaser (NHIF) and provider (healthcare facilities) factors continue to impair the responsiveness of the NHIF to the needs of individuals living with hypertension/diabetes. On the purchaser side, documented challenges included inadequate awareness about service entitlements in the NHIF Supa Cover package. Inadequate awareness of hypertension/diabetes service entitlements by healthcare workers and patients resulted in denial of care as patients would sometimes be turned away. A similar finding has been reported in a previous study [43]. In addition, respondents felt that the NHIF Supa Cover did not prioritise preventive and promotive services for NCDs but rather focused on curative services at secondary hospitals, that are not accessible to the rural poor. Nevertheless, targeted NCD screening is cost-effective [44-46] and there is evidence that the poor need NCD screening services more than the rich in Kenya [13].

Unaffordability of the NHIF Supa Cover monthly premiums and the penalties of defaulting payment were among the key weaknesses identified by study participants. This was specifically a challenge for enrolees from the informal sector, a finding that has been reported in previous studies conducted in Ghana and Kenya [43, 47, 48]. Furthermore, hypertension/diabetes patients enrolled in the NHIF Supa Cover from the informal sector were required to pay their monthly premium subscription at least one year in advance before NHIF provided authorisation for them to undergo costly or complex procedures. This presents a substantial financial barrier to patients and goes counter to the role of health insurance as a mechanism for pooling risks to avert the need to make large OOP payments when a health event occurs. Despite the NHIF providing some financial protection, OOP payments persisted. The unavailability of NCD medicines in public facilities has been corroborated by the findings of a previous study conducted in eight Kenyan counties [49]. Similarly, there is evidence of inequities in hypertension treatment in Kenya, with the poor being disproportionately disadvantaged [13].

Several purchaser and provider factors compromised physical access to NHIF-contracted facilities and quality of care for hypertension/diabetes services. First, except for inpatient admission, capitation-based payments by NHIF limited access to hypertension/diabetes services to only one NHIF-accredited provider. As such, patients had to pay OOP to access hypertension/diabetes services from other providers if the services they needed were unavailable from the providers where they were capitated. Second, infrastructural gaps hindered access and compromised the

For peer review only - http://bmjopen.bmj.com/site/about/guidelines.xhtml

quality of hypertension/diabetes services as patients were sometimes turned away and the required laboratory supplies and equipment were often broken down or lacking in most public facilities contracted by NHIF.

Whereas this study did not formally assess provider factors that influence service experience by individuals enrolled in the NHIF Supa Cover, there were provider factors that influenced respondents' experience of the Supa Cover benefit package. For instance, consistent with available literature [23, 50], some patients felt discriminated against by healthcare workers because they were enrolled in NHIF. Healthcare workers' negative attitudes towards NHIF enrolees is likely to lead to attrition [43]. This finding could be explained by NHIF's capitation-based provider payment mechanisms that are low and unpredictable [51].

This study had limitations. First, while we aimed to elicit experiences from hypertension/diabetes patients who were enrolled in the NHIF Supa Cover scheme at the time of the interview, including patients who had discontinued membership from NHIF could have yielded richer findings on NHIF responsiveness. Nevertheless, the findings from the quantitative component of our study that has included households that were enrolled into NHIF, discontinued their enrolment, or were not completely enrolled complements the findings of this qualitative work. Second, the study was limited to two counties, and a larger study across more counties may identify a greater range of experiences. However, the fact that the NHIF is a national agency, and the implementation of its programmes e.g., the benefit package or the premium contributions is standardized across geographies, gives confidence that observations in two countries are likely comparable to those in other counties.

Based on our study findings, we make the following policy recommendations. First, the NHIF should enhance its engagement and communication with its members living with hypertension/diabetes, health facilities and county managers to improve awareness about the entitlements in the NHIF benefit package. Second, the NHIF should review its benefit package to align with the needs of people living with hypertension/diabetes. This includes considering the inclusion of key diagnostic, and monitoring services for hypertension/diabetes, and expanding priority to include preventive and promotive services which are more cost-effective. Third, the NHIF should review the design of the outpatient capitation model to make provisions for portability. That is, the capitation mechanism should provide for individuals to access services in facilities that they are not capitated to in instances when this is necessary (such as when they travel or in case of an emergency). Fourth, the NHIF and county governments should strengthen monitoring and accountability of health facilities to address undesired provider behaviour such as

For peer review only - http://bmjopen.bmj.com/site/about/guidelines.xhtml

discrimination. Fifth, to address the structural quality issues of health facilities, the NHIF and county governments should enhance monitoring. Further, the NHIF should enhance its facility selection mechanism to assure the selection of health facilities with the structural capacity to deliver high-quality services, while the county government and health facilities should prioritise investments to improve the structural capacity of public health facilities. Health facility managers also have a role in implementing internal mechanism to prevent and address undesired provider behaviour through monitoring and sanctions. Sixth, the national government should consider providing full or partial subsidies to enhance the affordability of the NHIF premiums in the informal sector. This should be accompanied by a review of the additional administrative charges to enrol with the NHIF. Lastly, the NHIF should review its pre-authorisation requirements to remove financial barriers. Specifically, the requirement for NHIF members from the informal sector to make long-term premium payments before accessing expensive procedures goes counter to the intention to provide financial risk protection through pooling.

Conclusion

While the NHIF has reduced financial barriers and enhanced access to care for people living with hypertension and diabetes in Kenya, challenges persist that compromise NHIF's responsiveness to the needs of these patients. As the government of Kenya embarks on the national roll out its national UHC programme, a window of opportunity exists to enhance NHIF's responsiveness to the needs of patients living with hypertension/diabetes. The responsibility to do this is shared among key actors like the NHIF, the national and county governments, as well as health facilities.

For peer review only

For peer review only - http://bmjopen.bmj.com/site/about/guidelines.xhtml

Acknowledgment: The authors wish to acknowledge the county health officials from Busia and Trans Nzoia, research assistants and study participants for enabling the successful implementation of this research study.

Contributorship Statement: PP, JHK, EN, VN, AM, AE and EB conceptualised the study and obtained funding. EB, VW, RO, RW and HK contributed to the methodological development and study design. HK, RM, RO, and VW supported participant recruitment and data collection. EB, RW and RO analysed and interpreted the data. RO prepared the first draft manuscript that was subsequently reviewed and revised by all the authors. All authors read and approved the final manuscript.

Funding statement: This work was supported by the UK Medical Research Council grant number MR/T023538/1. RO is funded by a Wellcome Trust International Masters award (#214622), and additional funds from a Wellcome Trust core grant awarded to the KEMRI-Wellcome Trust Research Program (#092654) supported this work.

Competing interests: None declared

Ethical considerations

This study was reviewed and approved by the Institutional Ethics and Research Committee of Moi University (IREC 0003586) and the London School of Hygiene and Tropical Medicine (17940).

Data sharing: All data relevant to the study are included in the article or uploaded as supplementary information.

For peer review only

REFERENCES

1. Naghavi, M., et al., *Global, regional, and national age-sex specific mortality for 264 causes of death, 1980–2016: a systematic analysis for the Global Burden of Disease Study 2016.* The Lancet, 2017. **390**(10100): p. 1151-1210.
2. World Health Organization, *Global status report on noncommunicable diseases 2014*. 2014, World Health Organization.
3. World Health Organization, *Noncommunicable diseases country profiles 2018.* . 2018: Geneva: World Health Organization; 2018. Licence: CC BY-NC-SA 3.0 IGO.
4. Gouda, H.N., et al., *Burden of non-communicable diseases in sub-Saharan Africa, 1990–2017: results from the Global Burden of Disease Study 2017.* The Lancet Global Health, 2019. **7**(10): p. e1375-e1387.
5. Ministry of Health, *Kenya STEPwise survey for non-communicable diseases risk factors 2015 report. Available at:* https://www.knbs.or.ke/kenya-stepwise-survey-for-non-communicable-diseases-risk-factors-2015-report/ *[Accessed 8 February 2022]*. 2015.
6. Watkins, D.A., et al., *Universal health coverage and essential packages of care.* 2018.
7. World Health Organization, *Health systems financing: the path to universal coverage. Geneva: World Health Organization.* 2012.
8. Ataklte, F., et al., *Burden of undiagnosed hypertension in sub-saharan Africa: a systematic review and meta-analysis.* Hypertension, 2015. **65**(2): p. 291-298.
9. International Diabetes Federation, *IDF Diabetes Atlas, 9th edn.* 2019: Brussels, Belgium: International Diabetes Federation.
10. Murphy, A., et al., *Inequalities in the use of secondary prevention of cardiovascular disease by socioeconomic status: evidence from the PURE observational study.* The Lancet Global Health, 2018. **6**(3): p. e292-e301.
11. Sozmen, K. and U. Belgin, *Socioeconomic inequalities in non-communicable diseases and self assessed health in Turkey.* Iranian journal of public health, 2014. **43**(6): p. 736.
12. Vellakkal, S., et al., *Are estimates of socioeconomic inequalities in chronic disease artefactually narrowed by self-reported measures of prevalence in low-income and middle-income countries? Findings from the WHO-SAGE survey.* J Epidemiol Community Health, 2015. **69**(3): p. 218-225.
13. Oyando, R., E. Barasa, and J.E. Ataguba, *Socioeconomic Inequity in the Screening and Treatment of Hypertension in Kenya: Evidence From a National Survey.* Frontiers in Health Services, 2022: p. 7.
14. Nugent, R., et al., *Investing in non-communicable disease prevention and management to advance the Sustainable Development Goals.* The Lancet, 2018. **391**(10134): p. 2029-2035.
15. Jamison, D.T., et al., *Global health 2035: a world converging within a generation.* The lancet, 2013. **382**(9908): p. 1898-1955.
16. Kazibwe, J., P.B. Tran, and K.S. Annerstedt, *The household financial burden of non-communicable diseases in low-and middle-income countries: a systematic review.* Health research policy and systems, 2021. **19**(1): p. 1-15.
17. El-Sayed, A.M., et al., *Does health insurance mitigate inequities in non-communicable disease treatment? Evidence from 48 low-and middle-income countries.* Health Policy, 2015. **119**(9): p. 1164-1175.
18. Wagner, A.K., et al., *Access to care and medicines, burden of health care expenditures, and risk protection: results from the World Health Survey.* Health policy, 2011. **100**(2-3): p. 151-158.
19. Fang, J., et al., *Insurance status among adults with hypertension—the impact of underinsurance.* Journal of the American Heart Association, 2016. **5**(12): p. e004313.
20. Barasa, E., et al., *Examining the level and inequality in health insurance coverage in 36 sub-Saharan African countries.* BMJ Global Health, 2021. **6**(4): p. e004712.

For peer review only - http://bmjopen.bmj.com/site/about/guidelines.xhtml

21. Ly, M.S., O. Bassoum, and A. Faye, *Universal health insurance in Africa: a narrative review of the literature on institutional models.* BMJ Global Health, 2022. **7**(4): p. e008219.
22. Barasa, E., et al., *Kenya National Hospital Insurance Fund Reforms: implications and lessons for universal health coverage.* Health Systems & Reform, 2018. **4**(4): p. 346-361.
23. Kabia, E., et al., *"We are called the et cetera": experiences of the poor with health financing reforms that target them in Kenya.* International Journal for Equity in Health, 2019. **18**(1): p. 98.
24. Tama, E., et al., *Examining the implementation of the free maternity services policy in Kenya: a mixed methods process evaluation.* International journal of health policy and management, 2018. **7**(7): p. 603.
25. Chuma, J., et al., *Reducing user fees for primary health care in Kenya: Policy on paper or policy in practice?* International journal for equity in health, 2009. **8**(1): p. 1-10.
26. Munge, K., et al., *A critical analysis of purchasing arrangements in Kenya: the case of the National Hospital Insurance Fund.* International journal of health policy and management, 2018. **7**(3): p. 244.
27. KNBS and ICF, *Kenya Demographic and Health Survey 2022. Key Indicators Report.* 2023: Nairobi, Kenya, and Rockville, Maryland, USA: KNBS and ICF.
28. World Bank, *The World Bank Kenya Profile:* *http://data.worldbank.org/country/kenya* *(Accessed February 17, 2022).* 2022.
29. Government of Kenya, *The Constitution of Kenya*. 2010: Nairobi: Government of Kenya.
30. Ministry of Health, *Kenya Health Policy 2014-2030*. 2014.
31. Government of Kenya, *Kenya Service Availablity and Readiness Assessment Mapping (SARAM) Report.* 2013, Ministry of Health: Nairobi, Kenya
32. Ministry of Health Kenya, *Kenya Health Sector Strategic and Investment Plan: Transforming Health: Accelerating Attainment of Universal Health Coverage.* 2014, Ministry of Health: Nairobi, Kenya.
33. Barasa, E., P. Nguhiu, and D. McIntyre, *Measuring progress towards sustainable development goal 3.8 on universal health coverage in Kenya.* BMJ global health, 2018. **3**(3): p. e000904.
34. Munge, K. and A.H. Briggs, *The progressivity of health-care financing in Kenya.* Health policy and planning, 2013. **29**(7): p. 912-920.
35. Ministry of Health, *Republic of Kenya. National Strategic Plan for the Prevention and Control of Non-Communicable Diseases 2021/22–2025/26. Available* *https://www.health.go.ke/wp-content/uploads/2021/07/Kenya-Non-Communicable-Disease-NCD-Strategic-Plan-2021-2025.pdf* *(Accessed 24 June 2022)*. 2021: Nairobi, Kenya
36. Manji, I., et al., *The revolving fund pharmacy model: backing up the ministry of health supply chain in western Kenya.* International Journal of Pharmacy Practice, 2016. **24**(5): p. 358-366.
37. Nolte, E., et al., *Scaling up the primary health integrated care project for chronic conditions in Kenya: study protocol for an implementation research project.* 2021.
38. KNBS, *Kenya population and housing census*. 2019.
39. Ministry of Health. *Kenya Master Health Facility List, 2020:* *https://open.africa/dataset/kenya-master-health-facility-list-2020* *(Accessed 01 March 2022)*. 2020.
40. Andale, T., et al., *Anthropometric measurements as predictors of hypertension in Busia, Vihiga, Trans Nzoia and Siaya counties of Western Kenya.* medRxiv, 2021: p. 2021.07. 07.21258941.
41. Hennink, M. and B.N. Kaiser, *Sample sizes for saturation in qualitative research: A systematic review of empirical tests.* Social Science & Medicine, 2022. **292**: p. 114523.
42. Braun, V. and V. Clarke, *Using thematic analysis in psychology.* Qualitative research in psychology, 2006. **3**(2): p. 77-101.
43. Barasa, E.W., et al., *Extending voluntary health insurance to the informal sector: experiences and expectations of the informal sector in Kenya.* Wellcome open research, 2017. **2**.

For peer review only - http://bmjopen.bmj.com/site/about/guidelines.xhtml

44. Howard, K., et al., *Cost-effectiveness of screening and optimal management for diabetes, hypertension, and chronic kidney disease: a modeled analysis.* Value in health, 2010. **13**(2): p. 196-208.
45. Hoerger, T.J., et al., *Screening for type 2 diabetes mellitus: a cost-effectiveness analysis.* Annals of Internal Medicine, 2004. **140**(9): p. 689-699.
46. Kahn, R., et al., *Age at initiation and frequency of screening to detect type 2 diabetes: a cost-effectiveness analysis.* The Lancet, 2010. **375**(9723): p. 1365-1374.
47. Agyepong, I.A., et al., *The "Universal" in UHC and Ghana's National Health Insurance Scheme: policy and implementation challenges and dilemmas of a lower middle income country.* BMC Health Services Research, 2016. **16**(1): p. 1-14.
48. Okungu, V.R. and D. McIntyre, *Does the informal sector in Kenya have financial potential to sustainably prepay for health care? Implications for financing universal health coverage in low-income settings.* Health Systems & Reform, 2019. **5**(2): p. 145-157.
49. Ashigbie, P.G., et al., *Availability and prices of medicines for non-communicable diseases at health facilities and retail drug outlets in Kenya: a cross-sectional survey in eight counties.* BMJ open, 2020. **10**(5): p. e035132.
50. Jehu-Appiah, C., et al., *Household perceptions and their implications for enrolment in the National Health Insurance Scheme in Ghana.* Health policy and planning, 2012. **27**(3): p. 222-233.
51. Obadha, M., et al., *Health care purchasing in Kenya: Experiences of health care providers with capitation and fee-for-service provider payment mechanisms.* The International Journal of Health Planning and Management, 2019. **34**(1): p. e917-e933.

For peer review only

Figure 1: Study conceptual framework

For peer review only

For peer review only - http://bmjopen.bmj.com/site/about/guidelines.xhtml

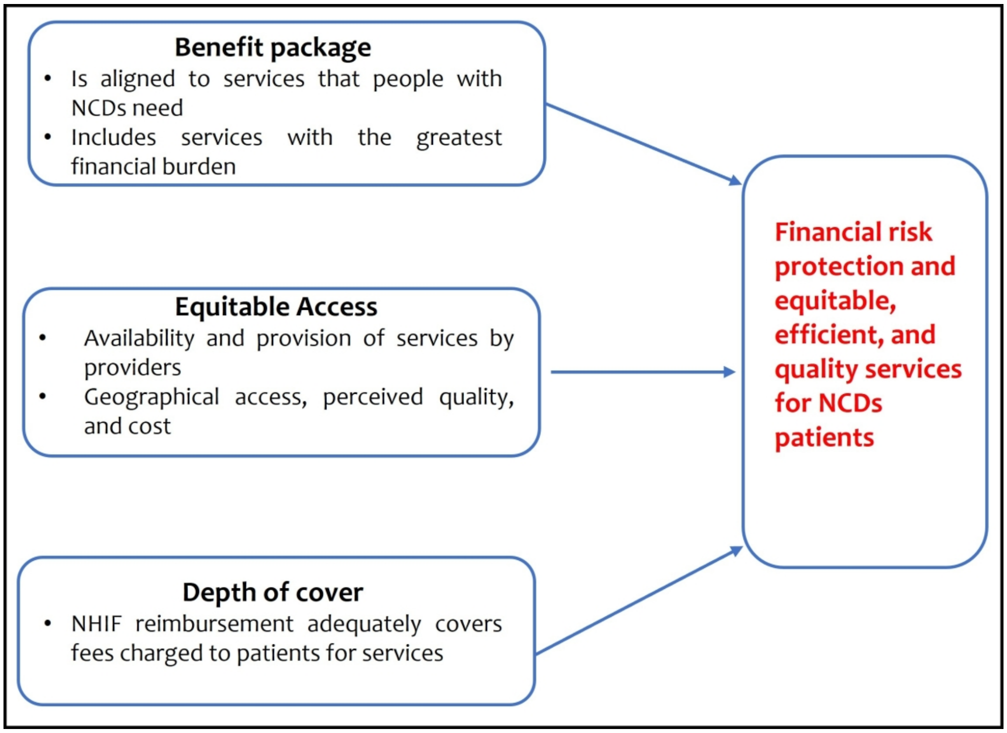

Figure 1 Study conceptual framework

173x125mm (330 x 330 DPI)

STROBE Statement—Checklist of items that should be included in reports of ***cross-sectional studies***

	Item No	Recommendation	Page No
**Title and abstract**	1	(*a*) Indicate the study's design with a commonly used term in the title or the abstract	1
		(*b*) Provide in the abstract an informative and balanced summary of what was done and what was found	2
**Introduction**			
Background/rationale	2	Explain the scientific background and rationale for the investigation being reported	3 to 5
Objectives	3	State specific objectives, including any prespecified hypotheses	
**Methods**			
Study design	4	Present key elements of study design early in the paper	7
Setting	5	Describe the setting, locations, and relevant dates, including periods of recruitment, exposure, follow-up, and data collection	6
Participants	6	(*a*) Give the eligibility criteria, and the sources and methods of selection of participants	7
Variables	7	Clearly define all outcomes, exposures, predictors, potential confounders, and effect modifiers. Give diagnostic criteria, if applicable	NA
Data sources/ measurement	8*	For each variable of interest, give sources of data and details of methods of assessment (measurement). Describe comparability of assessment methods if there is more than one group	NA
Bias	9	Describe any efforts to address potential sources of bias	8
Study size	10	Explain how the study size was arrived at	7 to 8
Quantitative variables	11	Explain how quantitative variables were handled in the analyses. If applicable, describe which groupings were chosen and why	NA
Statistical methods	12	(*a*) Describe all statistical methods, including those used to control for confounding	NA
		(*b*) Describe any methods used to examine subgroups and interactions	NA
		(*c*) Explain how missing data were addressed	NA
		(*d*) If applicable, describe analytical methods taking account of sampling strategy	NA
		(*e*) Describe any sensitivity analyses	NA
**Results**			
Participants	13*	(a) Report numbers of individuals at each stage of study—eg numbers potentially eligible, examined for eligibility, confirmed eligible, included in the study, completing follow-up, and analysed	10
		(b) Give reasons for non-participation at each stage	NA
		(c) Consider use of a flow diagram	NA
Descriptive data	14*	(a) Give characteristics of study participants (eg demographic, clinical, social) and information on exposures and potential confounders	10
		(b) Indicate number of participants with missing data for each variable of interest	NA
Outcome data	15*	Report numbers of outcome events or summary measures	NA

For peer review only - http://bmjopen.bmj.com/site/about/guidelines.xhtml

Main results	16	(*a*) Give unadjusted estimates and, if applicable, confounder-adjusted estimates and their precision (eg, 95% confidence interval). Make clear which confounders were adjusted for and why they were included	NA
		(*b*) Report category boundaries when continuous variables were categorized	NA
		(*c*) If relevant, consider translating estimates of relative risk into absolute risk for a meaningful time period	NA
Other analyses	17	Report other analyses done—eg analyses of subgroups and interactions, and sensitivity analyses	NA
**Discussion**			
Key results	18	Summarise key results with reference to study objectives	16
Limitations	19	Discuss limitations of the study, taking into account sources of potential bias or imprecision. Discuss both direction and magnitude of any potential bias	17
Interpretation	20	Give a cautious overall interpretation of results considering objectives, limitations, multiplicity of analyses, results from similar studies, and other relevant evidence	17
Generalisability	21	Discuss the generalisability (external validity) of the study results	17
**Other information**			
Funding	22	Give the source of funding and the role of the funders for the present study and, if applicable, for the original study on which the present article is based	18

*Give information separately for exposed and unexposed groups.

Note: An Explanation and Elaboration article discusses each checklist item and gives methodological background and published examples of transparent reporting. The STROBE checklist is best used in conjunction with this article (freely available on the Web sites of PLoS Medicine at http://www.plosmedicine.org/, Annals of Internal Medicine at http://www.annals.org/, and Epidemiology at http://www.epidem.com/). Information on the STROBE Initiative is available at www.strobe-statement.org.

For peer review only

BMJ Open

Examining the Responsiveness of the National Health Insurance Fund (NHIF) to People Living with Hypertension and Diabetes in Kenya: A qualitative study

Journal:	*BMJ Open*
Manuscript ID	bmjopen-2022-069330.R2
Article Type:	Original research
Date Submitted by the Author:	31-May-2023
Complete List of Authors:	Oyando, Robinson; KEMRI-Wellcome Trust Research Programme<br>Were, Vincent; KEMRI-Wellcome Trust Research Programme Nairobi, Health Economics Research Unit (HERU)<br>Willis, Ruth; London School of Hygiene and Tropical Medicine Faculty of Public Health and Policy, Department of Health Service Research and Policy<br>Koros, Hillary; Academic Model Providing Access to Healthcare<br>Kamano, Jemima; Moi University, Department of Medicine, School of Medicine, College of Health Sciences,<br>Naanyu, Violet; Moi University, School of Arts and Social Sciences; Moi University College of Health Sciences, Department of Medicine<br>Etyang, Anthony ; KEMRI-Wellcome Trust Research Programme, Department of Epidemiology and Demography<br>Mugo, Richard; AMPATH Kenya<br>Murphy, Adrianna; London School of Hygiene and Tropical Medicine Faculty of Public Health and Policy,<br>Nolte, Ellen; London School of Hygiene & Tropical Medicine, Department of Health Services Research and Policy<br>Perel, Pablo; LSMTM<br>Barasa, Edwine; Centre for Geographic Medicine Research Coast, Health Economics Research Unit
**Primary Subject Heading**:	Health economics
Secondary Subject Heading:	Qualitative research, Health economics, Public health, Health policy, Health services research
Keywords:	Hypertension < CARDIOLOGY, Health policy < HEALTH SERVICES ADMINISTRATION & MANAGEMENT, Health economics < HEALTH SERVICES ADMINISTRATION & MANAGEMENT, QUALITATIVE RESEARCH, PUBLIC HEALTH

SCHOLARONE™
Manuscripts

I, the Submitting Author has the right to grant and does grant on behalf of all authors of the Work (as defined in the below author licence), an exclusive licence and/or a non-exclusive licence for contributions from authors who are: i) UK Crown employees; ii) where BMJ has agreed a CC-BY licence shall apply, and/or iii) in accordance with the terms applicable for US Federal Government officers or employees acting as part of their official duties; on a worldwide, perpetual, irrevocable, royalty-free basis to BMJ Publishing Group Ltd ("BMJ") its licensees and where the relevant Journal is co-owned by BMJ to the co-owners of the Journal, to publish the Work in this journal and any other BMJ products and to exploit all rights, as set out in our licence.

The Submitting Author accepts and understands that any supply made under these terms is made by BMJ to the Submitting Author unless you are acting as an employee on behalf of your employer or a postgraduate student of an affiliated institution which is paying any applicable article publishing charge ("APC") for Open Access articles. Where the Submitting Author wishes to make the Work available on an Open Access basis (and intends to pay the relevant APC), the terms of reuse of such Open Access shall be governed by a Creative Commons licence – details of these licences and which Creative Commons licence will apply to this Work are set out in our licence referred to above.

Other than as permitted in any relevant BMJ Author's Self Archiving Policies, I confirm this Work has not been accepted for publication elsewhere, is not being considered for publication elsewhere and does not duplicate material already published. I confirm all authors consent to publication of this Work and authorise the granting of this licence.

For peer review only

Examining the Responsiveness of the National Health Insurance Fund (NHIF) to People Living with Hypertension and Diabetes in Kenya: A qualitative study

Robinson Oyando[1], Vincent Were[1], Ruth Willis[2], Hillary Koros[3], Jemima Kamano[4], Violet Naanyu[4,5], Anthony Etyang[6], Richard Mugo[3], Adrianna Murphy[2], Ellen Nolte[2], Pablo Perel[7], Edwine Barasa[1,8]

[1] Health Economics Research Unit, KEMRI-Wellcome Trust Research Programme, Nairobi, Kenya
[2] Department of Health Services Research and Policy, London School of Hygiene & Tropical Medicine, London, UK
[3]AMPATH, Eldoret, Kenya
[4] Department of Medicine, School of Medicine, College of Health Sciences, Moi University, Eldoret, Kenya
[5] School of Arts and Social Sciences, Moi University, Eldoret, Kenya
[6] Department of Epidemiology and Demography, KEMRI-Wellcome Trust Research Programme, Kilifi, Kenya
[7] Department of Non-Communicable Disease Epidemiology, London School of Hygiene and Tropical Medicine, London
[8] Center for Tropical Medicine and Global Health, Oxford University, Oxford, 01540, UK

Corresponding author:

Robinson Oyando
Health Economics Research Unit, KEMRI Wellcome Trust Research Programme,
P.O.BOX 43640-00100,
Nairobi, Kenya
Email: ROyando@kemri-wellcome.org

For peer review only

For peer review only - http://bmjopen.bmj.com/site/about/guidelines.xhtml

ABSTRACT

Objectives: To assess the responsiveness of the National Health Insurance Fund (NHIF) Supa Cover benefit package to the needs of individuals with diabetes and hypertension in Kenya.

Design, setting and participants: We carried out a qualitative study and collected data using key informant interviews (n = 39) and focus group discussions (n = 4) in two purposively selected counties in Western Kenya. Study participants were drawn from NHIF officials, county government officials, health facility managers, healthcare workers and individuals with hypertension and diabetes who were enrolled in NHIF. We analysed data using a thematic approach.

Results: Study participants reported that the NHIF Supa Cover benefit package expanded access to services for people living with hypertension and diabetes. However, the NHIF members and healthcare workers had inadequate awareness of the NHIF service entitlements. The NHIF benefit package inadequately covered the range of services needed by people living with hypertension and diabetes and the benefits package did not prioritise preventive and promotive services. Sometimes patients were discriminated against by healthcare providers who preferred cash-paying patients, and some NHIF-empanelled health facilities had inadequate structural inputs essential for quality of care. Study participants felt that the NHIF premium for the general scheme was unaffordable, and NHIF members faced additional out-of-pocket costs because of additional payments for services not available or covered.

Conclusion: Whereas NHIF has reduced financial barriers for hypertension and diabetes patients, to enhance its responsiveness to patient needs, NHIF should implement mechanisms to increase benefit package awareness among members and providers. In addition, preventive and promotive services should be included in NHIF's benefits package and mechanisms to monitor and hold contracted providers accountable should be strengthened.

Keywords: Universal health coverage, non-communicable diseases, reforms, Kenya, Low- and Middle-Income Countries

For peer review only - http://bmjopen.bmj.com/site/about/guidelines.xhtml

Strengths and limitations of this study

- This study used in-depth interviews and focus group discussions to comprehensively examine how responsive the national health insurance fund is to the healthcare needs of patients with hypertension and diabetes.
- The study recruited participants from both urban and rural sites and health staff from a range of cadres and thus provides evidence from a wide range of respondents with varied experiences.
- Including hypertension and diabetes patients who had discontinued membership from the national health insurance scheme could have yielded richer findings on the scheme's responsiveness.

INTRODUCTION

Non-communicable diseases (NCDs) account for the greatest share of the global disease burden and are one of the significant health challenges of the century [1, 2]. Low-and-middle income countries (LMICs) bear the highest NCD burden [3]. While infectious diseases contribute to the highest share of disease burden in sub-Saharan Africa (SSA), the burden of NCDs is rapidly increasing and is expected to grow [4]. In Kenya, NCDs account for 50% of inpatient admissions and 55% of hospital deaths [5]. Responsive health systems provide high-quality health services equitably and efficiently to the population in need without the risk of catastrophic or impoverishing healthcare expenditures [6, 7]. Evidence suggests that most NCD patients in LMICs do not have equal access to NCD services such as screening and treatment [8-13]. Among other things, this is due to lack of access due to financial barriers and inadequate capacity of health facilities to offer NCD care [8, 12-14].

One of the mechanisms to enable financial access to NCD services is designing and implementing pre-payment health financing mechanisms from tax or compulsory insurance contributions that provide financial risk protection to households and individuals living with NCDs and enhance access

For peer review only - http://bmjopen.bmj.com/site/about/guidelines.xhtml

to quality NCD services [15, 16]. Some evidence suggests that having health insurance is beneficial in enabling access to NCDs services [17-19]. Nevertheless, health insurance coverage in SSA remains low, with only four out of 36 SSA countries having health insurance coverage of any type exceeding 20% [20]. In addition, tax-based pre-payment mechanisms are preferred as an equitable health financing mechanism in SSA given the context of a large informal sector with a low contributory capacity [21].

Kenya has implemented several health financing initiatives [22-25]. One such initiative is the policy decision to use the National Health Insurance Fund (NHIF) as one of the key pre-payment health financing mechanisms [26]. NHIF is a public insurer in Kenya that was established in 1966 to provide mandatory health insurance to all Kenyans [22]. However, national NHIF coverage remains low, at 24% [27]. In the past 11 years, several reforms have been introduced in NHIF in an effort to enhance its capacity to deliver UHC [22]. Among these reforms is the broadening of the benefit package ('Supa Cover') of its general scheme (that targets all Kenyans) to include outpatient and specialised services for NCDs [22].

There is limited evidence about the degree to which the expanded NHIF national scheme is addressing the needs of people with hypertension and diabetes—two major NCDs in Kenya. This study aims to generate evidence that would be relevant in informing policymakers and other stakeholders on how the configuration of health insurance reforms can be tailored to address the needs of NCD patients and thus contribute to the achievement of international benchmarks for NCDs spelt out in Sustainable Development Goal 3.4.

METHODS

Study setting

Kenya is a lower-middle income (LMIC) country located in the East Africa region [28]. There are two levels of administration in Kenya's health system: national and county [29]. The national government (Ministry of Health) is responsible for policy and regulation in the health system while the 47 county governments are responsible for service delivery [30]. The Kenya health system is pluralistic, with an almost equal share of service provision by the public and private providers [31].

Kenya's health system has a four-tiered structure. Tier 1 is at the community level where community-based healthcare demand creation activities are undertaken as stipulated in the community health strategy; Tier 2 is at the primary care level where preventive and promotive services are provided in dispensaries and health centres; Tier 3 consists of both primary and

For peer review only - http://bmjopen.bmj.com/site/about/guidelines.xhtml

secondary referral hospitals; and Tier 4 consists of tertiary referral hospitals under the direct management of the Ministry of Health (MoH) [32].

The sources of health financing in Kenya include out-of-pocket (OOP) payments by households, government (national and county) tax revenues, donors, and member contributions from NHIF and private insurance companies [33, 34]. For instance, in the financial year (FY) 2015/16, current health expenditure (CHE) as a percentage of gross domestic product was 5.2%, CHE per capita in US$ was 78.6 while the proportion of CHE financed through OOP payments was 26.1% [33]. In addition, the total health expenditure (THE) on NCDs as a proportion of THE was 11% in FY 2017/18 [35].

For peer review only

For peer review only - http://bmjopen.bmj.com/site/about/guidelines.xhtml

Study sites

This study was carried out in Busia and Trans Nzoia counties in Western Kenya, where Moi University, Moi Teaching and Referral Hospital through AMPATH (Academic Model Providing Access to Healthcare) have partnered for several years with county governments to strengthen health systems across various care levels [36].

This study is part of a larger study that seeks to inform and support the scale-up of the Primary Health Integrated Care Project for Chronic Conditions (PIC4C) model for integrated management of people with hypertension, diabetes, and breast and cervical cancers in Kenya [37]. Alongside other work packages in the larger study, our work package had three overarching objectives: 1) to measure the effectiveness of the NHIF national scheme benefit package to provide financial risk protection to individuals with hypertension/diabetes; 2) to examine the extent to which the NHIF national scheme benefit package is responsive to the needs of individuals with hypertension/diabetes; and 3) to examine how the provider incentives generated by provider payment arrangements of the NHIF national scheme benefit package influence equity, efficiency, and quality of care. Our engagement with policy makers on the findings of the entire study included presentation of findings from both the quantitative (objective 1) and qualitative (objectives 2 and 3) work. However, the focus of this paper is on objective 2.

Table 1 outlines the key characteristics of the two counties. Launched in 2018, the PIC4C model was implemented by a partnership between AMPATH/Moi, the MoH and the World Bank and included (i) early case finding of people with hypertension, diabetes, cervical/ breast cancer at service level 1; (ii) structured referral to service providers at level 2 for confirmation of diagnosis and treatment initiation or referral to level 3 or 4 using structured protocols; (iii) initiation of treatment using structured treatment protocols and decision support tools at levels 2, 3 and 4; (iv) retention of patients in care supported by ongoing training of health workers at all care levels; (v) monitoring and evaluation supported by a health information system; and (vi) advising patients in care to enrol in the NHIF national scheme for sustainable health financing [37].

For peer review only - http://bmjopen.bmj.com/site/about/guidelines.xhtml

Table 1 Characteristics of the study setting

Characteristics	Busia county	Trans Nzoia county
Population‡	893,681	990,341
% Adults (18+ years) ‡	52%	53%
% Females ‡	52%	51%
Life expectancy at birth‡‡	47 years	60.5 years
Poverty level‡‡	64%	50%
Estimated hypertension prevalence*	23.1%	33.6%
Estimated diabetes prevalence‡‡	1.5%	2%
NHIF coverage‡‡	31%	20%
Number of health facilities†	184 community units served by 47 dispensaries, 12 health centers, 5 sub-county and 1 county hospital	198 community units served by 38 dispensaries, 8 health centers, 6 sub-county health facilities and 1 county hospital
Number of patients with selected NCDs currently being serviced in AMPATH sites‡‡	6,060 patients with hypertension, 1,113 with diabetes and 200 with cervical cancer.	4,375 patients with hypertension, and 2,089 with diabetes.
Number of facilities where the PIC4C pilot was implemented‡‡	40 facilities (5 subcounty hospitals, 12 health centers and 23 dispensaries).	33 facilities (6 subcounty hospitals, 8 health centers and 19 dispensaries).

(Sources: ‡‡[37];‡[38]; †[39];*[40])

Study conceptual framework

This study's conceptual framework (Figure 1) assumes that for the NHIF to be effective in providing financial risk protection to hypertension/diabetes patients and their households while enhancing access to quality health services, it is imperative that 1) the benefit package represents services that patients need, 2) that patients actually access the entitlements from the benefit package, and 3) the NHIF reimbursements to healthcare providers adequately covers the fees providers charge patients for services (depth of cover). Therefore, following the study's conceptual framework, responsiveness in this study is conceptualised to mean that the NHIF Supa Cover meets the needs of diabetes and hypertension patients in each of the three elements.

Insert Figure 1 here.

For peer review only - http://bmjopen.bmj.com/site/about/guidelines.xhtml

Study design and data collection

We conducted a qualitative study where we interviewed purposively selected participants. We conducted four focus group discussions (FGDs) with diabetes and hypertension patients in Busia (1 rural and 1 urban) and Trans Nzoia (1 rural and 1 urban) counties who were enrolled in the NHIF national scheme. FGD participants were selected from an existing PIC4-C scale-up study database (matched cohort wave 2) [37]. FGD participants were eligible if 1) they were actively enrolled in NHIF at the time of recruitment, 2) they had either diabetes or hypertension or comorbid (both diabetes and hypertension), 3) were more than 18 years of age, and 4) if they consented to participate in the study. Patients were purposively selected to ensure a range of age, gender, condition/s, and attendance at urban/rural facility. Eligible participants were invited to the FGD sessions by telephone. FGD sessions took place at the nearest public health facility and at a time convenient to the participants. In-depth interviews (IDI) participants were purposively selected to include a range of cadres in both counties. IDIs were held with county health officials (n = 7), county NHIF branch officers (n = 3), health facility managers (n = 12), and front-line healthcare workers (n = 17) composed of medical officers, pharmacists, nursing officers and clinical officers drawn from public providers (health centres [n=2], sub-county hospitals [n=3] and county hospital [n=1]) and private providers (faith-based facility [n=1] and health centre [n=1]). All study participants were consented and signed informed consent forms before participating in the interview sessions. FGD and IDI sessions were facilitated by two trained qualitative researchers. The FGDs were facilitated in Kiswahili while the IDIs were conducted in English. With consent from the participants, the sessions were audio recorded and complimentary notes were taken. FGDs lasted for an average of 48 minutes while the interviews lasted for an average of 35 minutes. Table 2 outlines the number of respondents interviewed by their respective categories.

For peer review only

For peer review only - http://bmjopen.bmj.com/site/about/guidelines.xhtml

Table 2 Summary of Respondents

County	Respondent	No. interviewed
Busia	County officials	4
	NHIF branch officers	1
	Facility managers	6
	Frontline healthcare workers	10
	NCD patient FGDs	2 (1 urban [7 participants]; 1 rural [6 participants])
Trans Nzoia	County officials	3
	NHIF branch officers	2
	Facility managers	6
	Frontline healthcare workers	7
	NCD patient FGDs	2 (1 urban [7 participants]; 1 rural [5 participants])

The FGDs and IDIs were conducted using semi-structured topic guides. The development of the questions in the discussion and interview guides was informed by the study's conceptual framework (Figure 1). The questions focused on hypertension/diabetes services that NHIF pays for, adequacy of the range of services covered by NHIF, participants' experience with service provision, quality of care, prioritisation of preventive and promotive services in the NHIF Supa cover scheme, NHIF provider payment mechanisms, strengths, and weaknesses of the NHIF national scheme. At the end of each session, the facilitator and note taker held debrief sessions which facilitated improving the interviewing process and revision of some questions to enhance clarity and inclusion of emerging themes that needed further exploration. Data collection was discontinued when data saturation was attained (i.e., when no new information was forthcoming). The total sample size (39 interviews and 4 FGDs) meets the recommendation for sample size (9-17 for interviews and 4-8 for FGDs) for qualitative studies to achieve saturation [41]. Data were collected between June and July 2021.

Data management and analysis

Qualitative FGDs and IDIs audio recordings were transcribed verbatim and translated to English where necessary. To validate and familiarise ourselves with the transcribed transcripts, we read and re-read the transcripts and ensured correctness by re-listening to sections of the audio recordings. Validated transcripts were imported into NVivo QSR 12 for coding and analysis. We used a thematic approach to analyse the qualitative data. Thematic analysis involved familiarisation with

For peer review only - http://bmjopen.bmj.com/site/about/guidelines.xhtml

the data through reading and re-reading the transcripts to identify emerging ideas, meanings, patterns and potential themes [42]. In the first stage of analysis, two authors (RO and RW) working independently coded the first few transcripts to identify key themes and developed the initial coding framework. This involved the generation of initial codes, grouping developed codes into descriptive themes by identifying matching patterns. Thereafter, a discussion among three authors (RO, RW and EB) was held where individual codes were compared, and a final coding framework was arrived at by consensus. Whereas the coding of the transcripts was guided by the study's conceptual framework, as data coding progressed iteratively, the coding framework was updated based on the emerging codes. In the next step, RO charted the data. During charting, emerging themes are identified through a process where coded data is reorganised. In this stage, RO summarised the ideas from each code with illustrative quotes from the data. EB checked and refined coding charts developed by RO. In the final phase, summaries from each thematic heading were critically interpreted and synthesised to identify key policy-relevant findings.

Trustworthiness

We built trustworthiness in the study findings by interviewing multiple participants to identify different perspectives (data source triangulation), using different methods of data collection (method triangulation), iterative questioning through rephrasing of questions, use of probes, holding peer debriefing sessions with the study team and member checking where we shared preliminary findings with participants and asked them to verify the resonance of our findings with their perspectives [43]. We ensured analytical rigor by using multiple coders; our coding team included an experienced sociologist (RW) as well as experienced health systems researchers (RO & EB) [43].

Reflexivity

All authors have conducted health systems research on non-communicable diseases across different LMICs. Specifically, three authors have previously conducted research on the economic burden of hypertension and diabetes in Kenya which influenced their interest in the study topic and methodology including sampling of participants, data collection methods and data analysis.

Patient and public involvement statement

The roll out of the PIC4C model in the two counties was accomplished by involving village chiefs and elders in the process. Patients and members of the public were not involved in the development of the proposal, or the design of the research presented here.

For peer review only - http://bmjopen.bmj.com/site/about/guidelines.xhtml

RESULTS

We conducted a total of 39 IDIs (17 men and 22 women) and four FGD sessions with 28 participants (15 women and 13 men). Six FGD participants had diabetes, 13 had hypertension and nine had both diabetes and hypertension. The results are presented under five key thematic areas: access to hypertension/diabetes services, awareness of hypertension/diabetes service entitlements in the NHIF Supa Cover scheme, adequacy of the range of hypertension/diabetes services covered by NHIF, provider payment mechanisms for hypertension/diabetes services and affordability of NHIF Supa Cover scheme. Summaries of key findings are provided under each thematic area.

Access to Hypertension/Diabetes Services

The NHIF benefit package expanded access to services for people living with hypertension/diabetes by reducing financial barriers. Several participants reported that the NHIF package had facilitated access to a range of hypertension/diabetes care services that they would otherwise have had to pay for OOP. Services that were reported to be covered included inpatient care and referral to NHIF accredited healthcare facilities, and medications. For example, one patient noted:

"When I have the NHIF, I have hope that I can go to the hospital even if I don't have any money and I can get medication compared to the one who does not have the NHIF." Rural FGD Participant, County A

One healthcare worker specifically related NHIF coverage to increased clinic attendance:

"The strength of NHIF super cover is that it has alleviated the suffering of these patients. There are patients who had stopped coming to the clinic because of financial challenges, but with the NHIF cover, we found that almost all our patients are always coming for the clinic… those who are covered are always coming for the clinic without fail." Healthcare worker, Sub-County Hospital, County A

Being enrolled in NHIF was reported to have beneficial implications for patients' wider financial situations, for example, avoiding the need to borrow money or sell assets to pay for health care services.

"It prevents you from borrowing and reduces stress for you and even respect comes. It prevents you from selling the farm, animals and any other thing." Rural FGD Participant, County B

Further, financial barriers were reduced for all population groups because the NHIF did not limit the enrolment of individuals on age or pre-existing NCD condition, unlike private health insurance.

For peer review only - http://bmjopen.bmj.com/site/about/guidelines.xhtml

Awareness of Hypertension/Diabetes service entitlements

Some NHIF members and healthcare workers had inadequate awareness of the NHIF service entitlements. While some study respondents showed awareness of the hypertension/diabetes care services that are covered by the NHIF Supa Cover scheme, others indicated that they were not adequately aware of service entitlements under the scheme.

"If you were to get a patient who needed radiotherapy or chemotherapy in my facility, we don't offer those services. I don't know if NHIF caters for that. I don't know to what extent… it does not cover some services such as chemotherapy or radiotherapy. I don't know if it caters for them, am not sure. I hope it does." Healthcare worker, Sub-County Hospital, County A

Inadequate awareness of hypertension/diabetes service entitlements in the Supa Cover scheme resulted in some patients being wrongfully turned away by healthcare workers.

"I asked if the NHIF card can be used to do x-ray and whoever was there told me that it cannot, you have to go outside the hospital. I didn't go and that is when I realised that the NHIF can do x-ray. That is when I realised that there are others who get services while others do not. The last time there was a nurse who came and asked on my behalf and she was told that it was working, and I got the services." Urban FGD Participant, County B

Adequacy of the range of hypertension/diabetes services

The NHIF benefit package inadequately covered the range of services needed by people living with hypertension and diabetes. While the cover catered for outpatient consultation and inpatient admissions, there was a strong perception among most NHIF members and healthcare workers that the NHIF Supa Cover package did not adequately cover the range of diagnostic, monitoring, and medicines that they needed and did not explicitly cover hypertension/diabetes medicines. Several participants reported incidents of relevant services having to be covered OOP by service users themselves.

"One day I came here and they sent me to go get an x-ray. When I got there, I was told that they cannot cover for that expense. "Go out". When I got out there, I asked and was told KES 5,200/= to get the x-ray. I was perplexed." Urban FGD, County A

"First thing urinalysis, HbA1c [glycated haemoglobin] *and UEC* [urea, electrolytes and creatinine]. *UEC will guide us whether to start with insulin oral or metformin alone. Then afterwards you need to follow up on this patient after three months you need HbA1c, but you find most of our clients mostly*

For peer review only

those relying on insurances, they're not able to pay so I can comfortably say they are not adequately covered using NHIF." Medical Officer, Private Hospital, County A

The NHIF benefit package did not prioritise preventive and promotive services for hypertension/diabetes. Some study respondents felt that the NHIF Supa Cover does not prioritise preventive and promotive services such as screening for NCDs but instead prioritises curative care after a patient is diagnosed with hypertension or diabetes. It was further felt that the NHIF prioritised secondary and admission care in hospitals rather than primary care services at lower-level facilities.

"The Supa Cover mostly deals with curative aspects of treatment. So, it doesn't deal with preventive aspects of treatment. So, it waits for a client to fall sick, then they can get services. There is no such thing as a preventive service, maybe going for annual check-up unless of course, you are covered under the civil servant type of cover where you have access to one check-up per year." Facility Manager, Sub-County Hospital, County A

"We were told that NHIF is able to cater for health checks, routine screening. And unfortunately, that one is not being done for everybody; it is not for the supa cover, it is for these other packages." County Official, County B

Provider payment mechanisms for hypertension/diabetes services

The design of the outpatient capitation payment mechanism reduced geographical access to services. The capitation payment mechanism required patients to be registered to a specific health facility for outpatient services. Some participants mentioned that this limited access to outpatient services when patients travelled far from their registered facility, and hence compromised continuity of care.

"The government should look into that… [so that] *if you have NHIF, you can receive treatment anywhere. It is not a must that one chooses a hospital to receive treatment."* Rural FGD Participant, County A

Quality of hypertension/diabetes care

NHIF patients were sometimes discriminated against by healthcare providers. Some study participants felt that cash-paying patients received more attention and care from healthcare workers than hypertension/diabetes patients enrolled in NHIF. For instance, respondents living

For peer review only - http://bmjopen.bmj.com/site/about/guidelines.xhtml

with hypertension/diabetes observed that healthcare workers did not give them the support they required when in a health facility once they mentioned that they had NHIF cards.

"When you walk in the corridors and meet a doctor and you tell them you have NHIF card, they look at you differently. It is like people see you as desperate because you are mentioning that card. I think they prefer cash because if you say you have the card, they don't want to assist you." Urban FGD Participant, County B

"There are times when you waste time walking around the hospital with no help until you decide to use cash instead of wasting the whole day. Sometimes they have a negative attitude if you talk to them about the card." Urban FGD Participant, County B

However, other participants had dissenting views as they reported that there was no difference in how healthcare workers handled hypertension/diabetes patients enrolled in NHIF and those who are not.

"No favouritism is there because one is using NHIF and the other one is using cash." Rural FGD Participant, County B

Some patients related how they were sometimes denied the healthcare services they needed because they had an NHIF card. These patients were instead asked to seek services from private providers.

"I was just given nifedipine alone for blood pressure. I had my fellow patient who was given all the drugs because she paid cash. She was given all drugs. I left there asking myself what the card had done. I was discharged and told to go and buy drugs. I was given only one drug." Urban FGD Participant, County B

Some NHIF-empanelled health facilities had an inadequate structural quality of care. This was characterised by overcrowding at the clinics, erratic supply of hypertension/diabetes medicines, diagnostic supplies and equipment. Some participants reported that they had to return on another clinic day or seek services in other (often private) health facilities and incur OOP.

"Again, after you've come for clinic, there is a schedule, and patients are many. You are told that time is up for receiving your patient book. Yet you got delayed at the laboratory. I have seen others being returned. Their book gets rejected." Urban FGD Participant, County A

For peer review only - http://bmjopen.bmj.com/site/about/guidelines.xhtml

"The challenge that is there, it is like there are some tests that they don't do. Like today I did ECG [electrocardiogram], to know the condition of my heart because the other day I came to the hospital. I told the doctor that I had pain that I could not understand. Then I was told to come and do ECG. The machine for ECG was spoilt and because I wanted the service, I had to go outside the hospital, and I paid." Urban FGD Participant, County B

Some patients and healthcare workers reported that public health facilities often did not provide inpatient diets that were suitable for patients with hypertension and diabetes. The meals they were served during inpatient admission in public facilities were contrary to the dietary advice they were given to manage their conditions.

"If they prepare uji (porridge), it has a lot of sugar. And you have been told not to take sugar. Who will prepare yours now? If they knew that, they would set apart a ward." Rural FGD Participant, County A

Affordability and Financial Risk Protection

Study respondents felt that the NHIF premiums for the general scheme were unaffordable. The KES 500 per month premium was deemed to be beyond reach by some respondents, especially in rural areas. As a result, people living with hypertension/diabetes could not keep up with the premium payments and regularly defaulted.

"The NHIF should reduce the premiums since the people in the rural areas see KES 500 as a lot of cash and fail to register or remain active." Rural FGD Participant, County B

Furthermore, individuals from the informal sector (whose membership was voluntary) who needed to undergo costly or complicated procedures were required to pay their monthly premium subscription at least one year in advance before NHIF authorised them to undergo any procedure. This was deemed unaffordable by many respondents.

"NHIF members from the informal sector are normally required to pay premiums at least more than 2 years in advance, depending on the cost of the procedure that the person is required to do. They are required to pay premiums for 2 years or more before the preauthorisation can be approved." Healthcare worker, FBO, County A

For peer review only - http://bmjopen.bmj.com/site/about/guidelines.xhtml

"If you're a NHIF contributor and self-employed, you pay the KES 500 package every month. You cannot get the surgery done unless you get the pre-authorised form, and you should have paid premiums upfront for 1 year for the surgery to be approved. You will find that most of these patients are old, some of them are unemployed so while they can pay KES 500 per month, it becomes a very big issue to pay for a year or more." County Official, County A

NHIF members faced additional out-of-pocket costs because of administration charges, input supply shortages, balance billing, and payments for services not covered. Due to supply-side inefficiencies, some patients reported that they were required to make OOP to purchase consumables like gloves or photocopying fees for receipts.

"Everyone paying for NHIF is struggling. Like the other day on Wednesday. The day has come, we get there, KES 10/= for photocopy and KES 50/= for gloves." Rural FGD Participant, County B

Further, the reimbursements NHIF paid providers for inpatient and outpatient services were deemed insufficient to cover the cost of offering the services. Hence, patients had to make additional payments to bridge the gap in service delivery costs.

"Per day NHIF payment for inpatient is KES 1,500, while outpatient capitation is KES 1,200 so they can actually surpass. In a day the bill can actually go beyond that because we are looking at for example cost of insulin, the cost of the drugs they are getting… So, in as much as NHIF would pay the KES 1,200, now the patient has to top up, yes." Facility Manager, County Referral Hospital, County A

Services that NHIF did not cover included dental services, eye examination, physiotherapy, x-ray and laboratory tests such as glycated haemoglobin test (HbA1c), liver or kidney function tests, urea, electrolyte, creatinine (UECs), among others.

"… when you've been sent to physiotherapy. They will tell you to add a certain amount of money that they will tell you. They say that the card does not cover that." Urban FGD Participant, County A

"Some tests like for the diabetics, if you want to do an HbA1c, they have to pay cash. If they have to do a thyroid function test, they have to pay cash. So, there are some tests, even PSA [prostate-specific antigen]*, for the men with the NCD, they have to pay in cash. So, I have really never understood why, whereas when they go to NHIF they are told it is fully covered by NHIF."* Healthcare worker, County Referral Hospital, County B.

As a result of these additional costs, some patients were reported to skip clinic visits hence compromising continuity of care.

For peer review only - http://bmjopen.bmj.com/site/about/guidelines.xhtml

"First of all, he has issues with the fare to the facility. Reaching the facility, he has to be sent to go and do a RBS [random blood sugar] *or a FBS* [fasting blood sugar]*. That is an extra cost. From there he has been told to go buy drugs for 3 months and he is unable to even buy worth one week. So, he might end up absconding from clinic or just stopping the care completely."* County Official, County A

For peer review only

For peer review only - http://bmjopen.bmj.com/site/about/guidelines.xhtml

DISCUSSION

This study aimed to assess the responsiveness of the NHIF general scheme to the needs of individuals with hypertension and/or diabetes. Our findings are consistent with international evidence that having health insurance can enhance access to NCDs services [17-19]. Study respondents reported that having NHIF was critical in improving access to NCDs services. However, purchaser (NHIF) and provider (healthcare facilities) factors continue to impair the responsiveness of the NHIF to the needs of individuals living with hypertension/diabetes. On the purchaser side, documented challenges included inadequate awareness about service entitlements in the NHIF Supa Cover package. Inadequate awareness of hypertension/diabetes service entitlements by healthcare workers and patients resulted in denial of care as patients would sometimes be turned away. A similar finding has been reported in a previous study [44]. In addition, respondents felt that the NHIF Supa Cover did not prioritise preventive and promotive services for NCDs but rather focused on curative services at secondary hospitals, that are not accessible to the rural poor. Nevertheless, targeted NCD screening is cost-effective [45-47] and there is evidence that the poor need NCD screening services more than the rich in Kenya [13].

Unaffordability of the NHIF Supa Cover monthly premiums and the penalties of defaulting payment were among the key weaknesses identified by study participants. This was specifically a challenge for enrolees from the informal sector, a finding that has been reported in previous studies conducted in Ghana and Kenya [44, 48, 49]. Furthermore, hypertension/diabetes patients enrolled in the NHIF Supa Cover from the informal sector were required to pay their monthly premium subscription at least one year in advance before NHIF provided authorisation for them to undergo costly or complex procedures. This presents a substantial financial barrier to patients and goes counter to the role of health insurance as a mechanism for pooling risks to avert the need to make large OOP payments when a health event occurs. Despite the NHIF providing some financial protection, OOP payments persisted. The unavailability of NCD medicines in public facilities has been corroborated by the findings of a previous study conducted in eight Kenyan counties [50]. Similarly, there is evidence of inequities in hypertension treatment in Kenya, with the poor being disproportionately disadvantaged [13].

Several purchaser and provider factors compromised physical access to NHIF-contracted facilities and quality of care for hypertension/diabetes services. First, except for inpatient admission, capitation-based payments by NHIF limited access to hypertension/diabetes services to only one NHIF-accredited provider. As such, patients had to pay OOP to access hypertension/diabetes services from other providers if the services they needed were unavailable from the providers where they were capitated. Second, infrastructural gaps hindered access and compromised the

For peer review only - http://bmjopen.bmj.com/site/about/guidelines.xhtml

quality of hypertension/diabetes services as patients were sometimes turned away and the required laboratory supplies and equipment were often broken down or lacking in most public facilities contracted by NHIF.

Whereas this study did not formally assess provider factors that influence service experience by individuals enrolled in the NHIF Supa Cover, there were provider factors that influenced respondents' experience of the Supa Cover benefit package. For instance, consistent with available literature [23, 51], some patients felt discriminated against by healthcare workers because they were enrolled in NHIF. Healthcare workers' negative attitudes towards NHIF enrolees is likely to lead to attrition [44]. This finding could be explained by NHIF's capitation-based provider payment mechanisms that are low and unpredictable [52].

This study had limitations. First, while we aimed to elicit experiences from hypertension/diabetes patients who were enrolled in the NHIF Supa Cover scheme at the time of the interview, including patients who had discontinued membership from NHIF could have yielded richer findings on NHIF responsiveness. Nevertheless, the findings from the quantitative component of our study that has included households that were enrolled into NHIF, discontinued their enrolment, or were not completely enrolled complements the findings of this qualitative work. Second, the study was limited to two counties, and a larger study across more counties may identify a greater range of experiences. However, the fact that the NHIF is a national agency, and the implementation of its programmes e.g., the benefit package or the premium contributions is standardized across geographies, gives confidence that observations in two countries are likely comparable to those in other counties.

Based on our study findings, we make the following policy recommendations. First, the NHIF should enhance its engagement and communication with its members living with hypertension/diabetes, health facilities and county managers to improve awareness about the entitlements in the NHIF benefit package. Second, the NHIF should review its benefit package to align with the needs of people living with hypertension/diabetes. This includes considering the inclusion of key diagnostic, and monitoring services for hypertension/diabetes, and expanding priority to include preventive and promotive services which are more cost-effective. Third, the NHIF should review the design of the outpatient capitation model to make provisions for portability. That is, the capitation mechanism should provide for individuals to access services in facilities that they are not capitated to in instances when this is necessary (such as when they travel or in case of an emergency). Fourth, the NHIF and county governments should strengthen monitoring and accountability of health facilities to address undesired provider behaviour such as

For peer review only - http://bmjopen.bmj.com/site/about/guidelines.xhtml

discrimination. Fifth, to address the structural quality issues of health facilities, the NHIF and county governments should enhance monitoring. Further, the NHIF should enhance its facility selection mechanism to assure the selection of health facilities with the structural capacity to deliver high-quality services, while the county government and health facilities should prioritise investments to improve the structural capacity of public health facilities. Health facility managers also have a role in implementing internal mechanism to prevent and address undesired provider behaviour through monitoring and sanctions. Sixth, the national government should consider providing full or partial subsidies to enhance the affordability of the NHIF premiums in the informal sector. This should be accompanied by a review of the additional administrative charges to enrol with the NHIF. Lastly, the NHIF should review its pre-authorisation requirements to remove financial barriers. Specifically, the requirement for NHIF members from the informal sector to make long-term premium payments before accessing expensive procedures goes counter to the intention to provide financial risk protection through pooling.

Conclusion

While the NHIF has reduced financial barriers and enhanced access to care for people living with hypertension and diabetes in Kenya, challenges persist that compromise NHIF's responsiveness to the needs of these patients. As the government of Kenya embarks on the national roll out its national UHC programme, a window of opportunity exists to enhance NHIF's responsiveness to the needs of patients living with hypertension/diabetes. The responsibility to do this is shared among key actors like the NHIF, the national and county governments, as well as health facilities.

For peer review only

For peer review only - http://bmjopen.bmj.com/site/about/guidelines.xhtml

Acknowledgment: The authors wish to acknowledge the county health officials from Busia and Trans Nzoia, research assistants and study participants for enabling the successful implementation of this research study.

Contributorship Statement: PP, JHK, EN, VN, AM, AE and EB conceptualised the study and obtained funding. EB, VW, RO, RW and HK contributed to the methodological development and study design. HK, RM, RO, and VW supported participant recruitment and data collection. EB, RW and RO analysed and interpreted the data. RO prepared the first draft manuscript that was subsequently reviewed and revised by all the authors. All authors read and approved the final manuscript.

Funding statement: This work was supported by the UK Medical Research Council grant number MR/T023538/1. RO is funded by a Wellcome Trust International Masters award (#214622), and additional funds from a Wellcome Trust core grant awarded to the KEMRI-Wellcome Trust Research Program (#092654) supported this work.

Competing interests: None declared

Ethical considerations

This study was reviewed and approved by the Institutional Ethics and Research Committee of Moi University (IREC 0003586) and the London School of Hygiene and Tropical Medicine (17940).

Data sharing: All data relevant to the study are included in the article or uploaded as supplementary information.

For peer review only

For peer review only - http://bmjopen.bmj.com/site/about/guidelines.xhtml

REFERENCES

1. Naghavi, M., et al., *Global, regional, and national age-sex specific mortality for 264 causes of death, 1980–2016: a systematic analysis for the Global Burden of Disease Study 2016.* The Lancet, 2017. **390**(10100): p. 1151-1210.
2. World Health Organization, *Global status report on noncommunicable diseases 2014*. 2014, World Health Organization.
3. World Health Organization, *Noncommunicable diseases country profiles 2018.* . 2018: Geneva: World Health Organization; 2018. Licence: CC BY-NC-SA 3.0 IGO.
4. Gouda, H.N., et al., *Burden of non-communicable diseases in sub-Saharan Africa, 1990–2017: results from the Global Burden of Disease Study 2017.* The Lancet Global Health, 2019. **7**(10): p. e1375-e1387.
5. Ministry of Health, *Kenya STEPwise survey for non-communicable diseases risk factors 2015 report. Available at: https://www.knbs.or.ke/kenya-stepwise-survey-for-non-communicable-diseases-risk-factors-2015-report/ [Accessed 8 February 2022]*. 2015.
6. Watkins, D.A., et al., *Universal health coverage and essential packages of care.* 2018.
7. World Health Organization, *Health systems financing: the path to universal coverage. Geneva: World Health Organization.* 2012.
8. Ataklte, F., et al., *Burden of undiagnosed hypertension in sub-saharan Africa: a systematic review and meta-analysis.* Hypertension, 2015. **65**(2): p. 291-298.
9. International Diabetes Federation, *IDF Diabetes Atlas, 9th edn.* 2019: Brussels, Belgium: International Diabetes Federation.
10. Murphy, A., et al., *Inequalities in the use of secondary prevention of cardiovascular disease by socioeconomic status: evidence from the PURE observational study.* The Lancet Global Health, 2018. **6**(3): p. e292-e301.
11. Sozmen, K. and U. Belgin, *Socioeconomic inequalities in non-communicable diseases and self assessed health in Turkey.* Iranian journal of public health, 2014. **43**(6): p. 736.
12. Vellakkal, S., et al., *Are estimates of socioeconomic inequalities in chronic disease artefactually narrowed by self-reported measures of prevalence in low-income and middle-income countries? Findings from the WHO-SAGE survey.* J Epidemiol Community Health, 2015. **69**(3): p. 218-225.
13. Oyando, R., E. Barasa, and J.E. Ataguba, *Socioeconomic Inequity in the Screening and Treatment of Hypertension in Kenya: Evidence From a National Survey.* Frontiers in Health Services, 2022: p. 7.
14. Nugent, R., et al., *Investing in non-communicable disease prevention and management to advance the Sustainable Development Goals.* The Lancet, 2018. **391**(10134): p. 2029-2035.
15. Jamison, D.T., et al., *Global health 2035: a world converging within a generation.* The lancet, 2013. **382**(9908): p. 1898-1955.
16. Kazibwe, J., P.B. Tran, and K.S. Annerstedt, *The household financial burden of non-communicable diseases in low-and middle-income countries: a systematic review.* Health research policy and systems, 2021. **19**(1): p. 1-15.
17. El-Sayed, A.M., et al., *Does health insurance mitigate inequities in non-communicable disease treatment? Evidence from 48 low-and middle-income countries.* Health Policy, 2015. **119**(9): p. 1164-1175.
18. Wagner, A.K., et al., *Access to care and medicines, burden of health care expenditures, and risk protection: results from the World Health Survey.* Health policy, 2011. **100**(2-3): p. 151-158.
19. Fang, J., et al., *Insurance status among adults with hypertension—the impact of underinsurance.* Journal of the American Heart Association, 2016. **5**(12): p. e004313.
20. Barasa, E., et al., *Examining the level and inequality in health insurance coverage in 36 sub-Saharan African countries.* BMJ Global Health, 2021. **6**(4): p. e004712.

For peer review only - http://bmjopen.bmj.com/site/about/guidelines.xhtml

21. Ly, M.S., O. Bassoum, and A. Faye, *Universal health insurance in Africa: a narrative review of the literature on institutional models.* BMJ Global Health, 2022. **7**(4): p. e008219.
22. Barasa, E., et al., *Kenya National Hospital Insurance Fund Reforms: implications and lessons for universal health coverage.* Health Systems & Reform, 2018. **4**(4): p. 346-361.
23. Kabia, E., et al., *"We are called the et cetera": experiences of the poor with health financing reforms that target them in Kenya.* International Journal for Equity in Health, 2019. **18**(1): p. 98.
24. Tama, E., et al., *Examining the implementation of the free maternity services policy in Kenya: a mixed methods process evaluation.* International journal of health policy and management, 2018. **7**(7): p. 603.
25. Chuma, J., et al., *Reducing user fees for primary health care in Kenya: Policy on paper or policy in practice?* International journal for equity in health, 2009. **8**(1): p. 1-10.
26. Munge, K., et al., *A critical analysis of purchasing arrangements in Kenya: the case of the National Hospital Insurance Fund.* International journal of health policy and management, 2018. **7**(3): p. 244.
27. KNBS and ICF, *Kenya Demographic and Health Survey 2022. Key Indicators Report.* 2023: Nairobi, Kenya, and Rockville, Maryland, USA: KNBS and ICF.
28. World Bank, *The World Bank Kenya Profile: http://data.worldbank.org/country/kenya (Accessed February 17, 2022).* 2022.
29. Government of Kenya, *The Constitution of Kenya*. 2010: Nairobi: Government of Kenya.
30. Ministry of Health, *Kenya Health Policy 2014-2030*. 2014.
31. Government of Kenya, *Kenya Service Availablity and Readiness Assessment Mapping (SARAM) Report.* 2013, Ministry of Health: Nairobi, Kenya
32. Ministry of Health Kenya, *Kenya Health Sector Strategic and Investment Plan: Transforming Health: Accelerating Attainment of Universal Health Coverage.* 2014, Ministry of Health: Nairobi, Kenya.
33. Barasa, E., P. Nguhiu, and D. McIntyre, *Measuring progress towards sustainable development goal 3.8 on universal health coverage in Kenya.* BMJ global health, 2018. **3**(3): p. e000904.
34. Munge, K. and A.H. Briggs, *The progressivity of health-care financing in Kenya.* Health policy and planning, 2013. **29**(7): p. 912-920.
35. Ministry of Health, *Republic of Kenya. National Strategic Plan for the Prevention and Control of Non-Communicable Diseases 2021/22–2025/26. Available https://www.health.go.ke/wp-content/uploads/2021/07/Kenya-Non-Communicable-Disease-NCD-Strategic-Plan-2021-2025.pdf (Accessed 24 June 2022).* 2021: Nairobi, Kenya
36. Manji, I., et al., *The revolving fund pharmacy model: backing up the ministry of health supply chain in western Kenya.* International Journal of Pharmacy Practice, 2016. **24**(5): p. 358-366.
37. Nolte, E., et al., *Scaling up the primary health integrated care project for chronic conditions in Kenya: study protocol for an implementation research project.* 2021.
38. KNBS, *Kenya population and housing census*. 2019.
39. Ministry of Health. *Kenya Master Health Facility List, 2020: https://open.africa/dataset/kenya-master-health-facility-list-2020 (Accessed 01 March 2022).* 2020.
40. Andale, T., et al., *Anthropometric measurements as predictors of hypertension in Busia, Vihiga, Trans Nzoia and Siaya counties of Western Kenya.* medRxiv, 2021: p. 2021.07. 07.21258941.
41. Hennink, M. and B.N. Kaiser, *Sample sizes for saturation in qualitative research: A systematic review of empirical tests.* Social Science & Medicine, 2022. **292**: p. 114523.
42. Braun, V. and V. Clarke, *Using thematic analysis in psychology.* Qualitative research in psychology, 2006. **3**(2): p. 77-101.
43. O'Brien, B.C., et al., *Standards for reporting qualitative research: a synthesis of recommendations.* Academic medicine, 2014. **89**(9): p. 1245-1251.

For peer review only

For peer review only - http://bmjopen.bmj.com/site/about/guidelines.xhtml

44. Barasa, E.W., et al., *Extending voluntary health insurance to the informal sector: experiences and expectations of the informal sector in Kenya.* Wellcome open research, 2017. **2**.
45. Howard, K., et al., *Cost-effectiveness of screening and optimal management for diabetes, hypertension, and chronic kidney disease: a modeled analysis.* Value in health, 2010. **13**(2): p. 196-208.
46. Hoerger, T.J., et al., *Screening for type 2 diabetes mellitus: a cost-effectiveness analysis.* Annals of Internal Medicine, 2004. **140**(9): p. 689-699.
47. Kahn, R., et al., *Age at initiation and frequency of screening to detect type 2 diabetes: a cost-effectiveness analysis.* The Lancet, 2010. **375**(9723): p. 1365-1374.
48. Agyepong, I.A., et al., *The "Universal" in UHC and Ghana's National Health Insurance Scheme: policy and implementation challenges and dilemmas of a lower middle income country.* BMC Health Services Research, 2016. **16**(1): p. 1-14.
49. Okungu, V.R. and D. McIntyre, *Does the informal sector in Kenya have financial potential to sustainably prepay for health care? Implications for financing universal health coverage in low-income settings.* Health Systems & Reform, 2019. **5**(2): p. 145-157.
50. Ashigbie, P.G., et al., *Availability and prices of medicines for non-communicable diseases at health facilities and retail drug outlets in Kenya: a cross-sectional survey in eight counties.* BMJ open, 2020. **10**(5): p. e035132.
51. Jehu-Appiah, C., et al., *Household perceptions and their implications for enrolment in the National Health Insurance Scheme in Ghana.* Health policy and planning, 2012. **27**(3): p. 222-233.
52. Obadha, M., et al., *Health care purchasing in Kenya: Experiences of health care providers with capitation and fee-for-service provider payment mechanisms.* The International Journal of Health Planning and Management, 2019. **34**(1): p. e917-e933.

For peer review only

Figure 1: Study conceptual framework

For peer review only

For peer review only - http://bmjopen.bmj.com/site/about/guidelines.xhtml

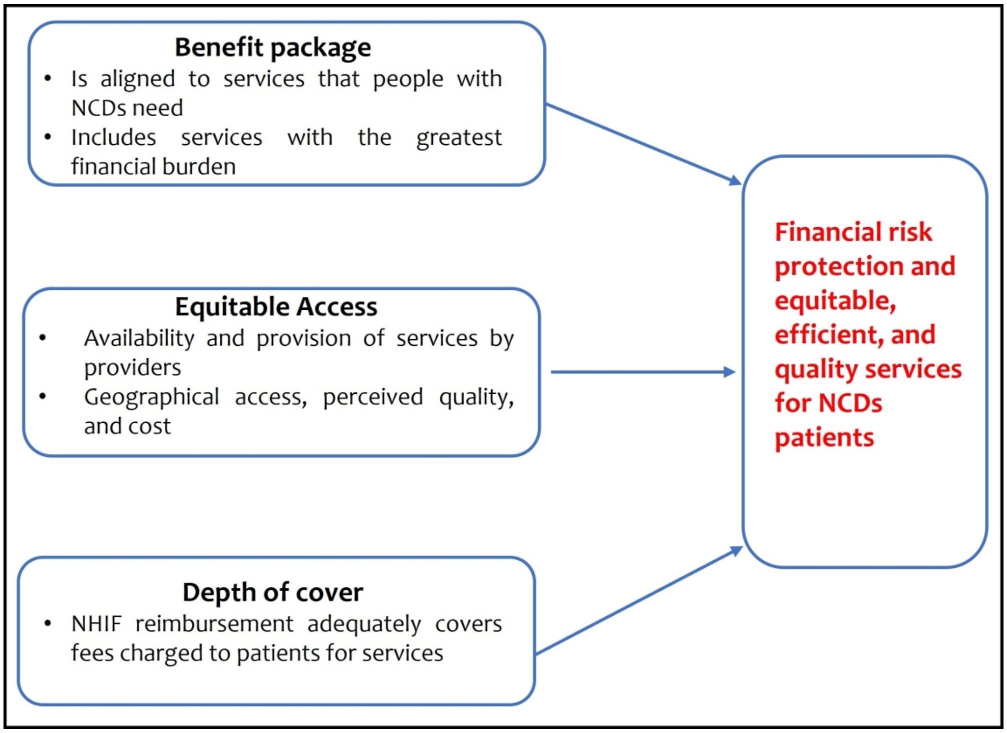

Figure 1 Study conceptual framework

173x125mm (330 x 330 DPI)

Standards for Reporting Qualitative Research

Topic	No.	Item	Page No
**Title**	S1	Concise description of the nature and topic of the study Identifying the study as qualitative or indicating the approach (e.g., ethnography, grounded theory) or data collection methods (e.g., interview, focus group) is recommended	Pg. 1, Line 1-3
**Abstract**	S2	Summary of key elements of the study using the abstract format of the intended publication; typically includes background, purpose, methods, results, and conclusions	Pg. 2, Line 28-49
**Introduction**			
Problem formulation	S3	Description and significance of the problem/phenomenon studied; review of relevant theory and empirical work; problem statement	Pg.3-4, Line 62-89
Purpose or research question	S4	Purpose of the study and specific objectives or questions	Pg. 4, Line 91-96
**Methods**			
Qualitative approach and research paradigm	S5	Qualitative approach (e.g., ethnography, grounded theory, case study, phenomenology, narrative research) and guiding theory if appropriate; identifying the research paradigm (e.g., postpositivist, constructivist/ interpretivist) is also recommended; rationale	Pg.6-7, Line 146-156 & Line 158-166
Researcher characteristics and reflexivity	S6	Researchers' characteristics that may influence the research, including personal attributes, qualifications/experience, relationship with participants, assumptions, and/or presuppositions; potential or actual interaction between researchers' characteristics and the research questions, approach, methods, results, and/or transferability	Pg. 9, Line 215-222
Context	S7	Setting/site and salient contextual factors; rationale	Pg.4, Line 99-116
Sampling strategy	S8	How and why research participants, documents, or events were selected; criteria for deciding when no further sampling was necessary (e.g., sampling saturation); rationale	Page 7-8; Line 166-187 & Line 190-194
Ethical issues pertaining to human subjects	S9	Documentation of approval by an appropriate ethics review board and participant consent, or explanation for lack thereof; other confidentiality and data security issues	Pg. 20, Line 538-540
Data collection methods	S10	Types of data collected; details of data collection procedures including (as appropriate) start and stop dates of data collection and analysis, iterative process, triangulation of sources/methods, and modification of	Pg. 7-8; Line 158-213

For peer review only

		procedures in response to evolving study findings; rationale	
Data collection instruments and technologies	S11	Description of instruments (e.g., interview guides, questionnaires) and devices (e.g., audio recorders) used for data collection; if/how the instrument(s) changed over the course of the study	Pg.7, Line 182-190
Units of study	S12	Number and relevant characteristics of participants, documents, or events included in the study; level of participation (could be reported in results)	Pg.10, Line 234-240
Data processing	S13	Methods for processing data prior to and during analysis, including transcription, data entry, data management and security, verification of data integrity, data coding, and anonymization/deidentification of excerpts	Pg. 8, Line 196-199
Data analysis	S14	Process by which inferences, themes, etc., were identified and developed, including the researchers involved in data analysis; usually references a specific paradigm or approach; rationale	Pg. 8-9, Line 199-213
Techniques to enhance trustworthiness	S15	Techniques to enhance trustworthiness and credibility of data analysis (e.g., member checking, audit trail, triangulation); rationale	Pg. 9, Line 215-222
**Results**			
Synthesis and interpretation	S16	Main findings (e.g., interpretations, inferences, and themes); might include development of a theory or model, or integration with prior research or theory	Pg. 10-16
Links to empirical data	S17	Evidence (e.g., quotes, field notes, text excerpts, photographs) to substantiate analytic findings	Pg. 10-16
**Discussion**			
Integration with prior work, implications, transferability, and contribution(s) to the field	S18	Short summary of main findings; explanation of how findings and conclusions connect to, support, elaborate on, or challenge conclusions of earlier scholarship; discussion of scope of application/ generalizability; identification of unique contribution(s) to scholarship in a discipline or field	Pg. 17 & 18, Line 437-479; Line 491-515
Limitations	S19	Trustworthiness and limitations of findings	Pg. 18, Line 480-490
**Other**			
Conflicts of interest	S20	Potential sources of influence or perceived influence on study conduct and conclusions; how these were managed	Pg. 20, Line 537
Funding	S21	Sources of funding and other support; role of funders in data collection, interpretation, and reporting	Pg. 20, Line 533-536

For peer review only
